# LncSEA: a platform for long non-coding RNA related sets and enrichment analysis

**DOI:** 10.1093/nar/gkaa806

**Published:** 2020-10-12

**Authors:** Jiaxin Chen, Jian Zhang, Yu Gao, Yanyu Li, Chenchen Feng, Chao Song, Ziyu Ning, Xinyuan Zhou, Jianmei Zhao, Minghong Feng, Yuexin Zhang, Ling Wei, Qi Pan, Yong Jiang, Fengcui Qian, Junwei Han, Yongsan Yang, Qiuyu Wang, Chunquan Li

**Affiliations:** School of Medical Informatics, Daqing Campus, Harbin Medical University, Daqing 163319, China; School of Medical Informatics, Daqing Campus, Harbin Medical University, Daqing 163319, China; School of Medical Informatics, Daqing Campus, Harbin Medical University, Daqing 163319, China; School of Medical Informatics, Daqing Campus, Harbin Medical University, Daqing 163319, China; School of Medical Informatics, Daqing Campus, Harbin Medical University, Daqing 163319, China; Department of Pharmacology, Daqing Campus, Harbin Medical University, Daqing 163319, China; School of Medical Informatics, Daqing Campus, Harbin Medical University, Daqing 163319, China; School of Medical Informatics, Daqing Campus, Harbin Medical University, Daqing 163319, China; School of Medical Informatics, Daqing Campus, Harbin Medical University, Daqing 163319, China; School of Medical Informatics, Daqing Campus, Harbin Medical University, Daqing 163319, China; School of Medical Informatics, Daqing Campus, Harbin Medical University, Daqing 163319, China; School of Medical Informatics, Daqing Campus, Harbin Medical University, Daqing 163319, China; School of Medical Informatics, Daqing Campus, Harbin Medical University, Daqing 163319, China; School of Medical Informatics, Daqing Campus, Harbin Medical University, Daqing 163319, China; School of Medical Informatics, Daqing Campus, Harbin Medical University, Daqing 163319, China; College of Bioinformatics Science and Technology, Harbin Medical University, Harbin 150081, China; School of Medical Informatics, Daqing Campus, Harbin Medical University, Daqing 163319, China; School of Medical Informatics, Daqing Campus, Harbin Medical University, Daqing 163319, China; School of Medical Informatics, Daqing Campus, Harbin Medical University, Daqing 163319, China

## Abstract

Long non-coding RNAs (lncRNAs) have been proven to play important roles in transcriptional processes and various biological functions. Establishing a comprehensive collection of human lncRNA sets is urgent work at present. Using reference lncRNA sets, enrichment analyses will be useful for analyzing lncRNA lists of interest submitted by users. Therefore, we developed a human lncRNA sets database, called LncSEA, which aimed to document a large number of available resources for human lncRNA sets and provide annotation and enrichment analyses for lncRNAs. LncSEA supports >40 000 lncRNA reference sets across 18 categories and 66 sub-categories, and covers over 50 000 lncRNAs. We not only collected lncRNA sets based on downstream regulatory data sources, but also identified a large number of lncRNA sets regulated by upstream transcription factors (TFs) and DNA regulatory elements by integrating TF ChIP-seq, DNase-seq, ATAC-seq and H3K27ac ChIP-seq data. Importantly, LncSEA provides annotation and enrichment analyses of lncRNA sets associated with upstream regulators and downstream targets. In summary, LncSEA is a powerful platform that provides a variety of types of lncRNA sets for users, and supports lncRNA annotations and enrichment analyses. The LncSEA database is freely accessible at http://bio.liclab.net/LncSEA/index.php.

## INTRODUCTION

Long noncoding RNAs (lncRNAs) play key roles in biological processes and can even be used as novel biomarkers (1–4). Mutations to, and the methylation of, lncRNAs may also affect lncRNA expression levels, leading to diseases such as cancer (5,6). In recent years, some lncRNAs defining cellular identity were discovered by biological experiments and single cell sequencing techniques (7,8). Many studies showed that the functions of lncRNAs are closely related to their location on the inside and outside the cell. For example exosomal lncRNA H19 could promote hepatic stellate cell activation and cholestatic liver fibrosis (9). A large number of studies showed that lncRNAs perform a variety of regulatory functions for downstream genes. Ulitsky *et al.* demonstrated that lncRNA H19 functions as a competing endogenous RNA by binding miR-17-5p family members in HeLa cells and myoblasts (10). LncRNAs also bind to proteins and localize protein complexes to specific DNA sequences, which affect gene expression and the development of disease. For example, the FOXN3–NEAT1–SIN3A repressor complex promotes the progression of hormonally-responsive breast cancer (11). A large number of recent studies focused on transcripts annotated as lncRNAs, but encoded small proteins (12,13). Furthermore, the genomes of many species are transcribed pervasively, producing many lncRNAs with unknown functions. Increasing evidence suggests that lncRNAs can be regulated by upstream transcriptional regulators, including transcription factors (TFs) and DNA regulatory elements such as promoters, enhancers, super enhancers (SEs), and accessible chromatin regions (14–17). For example, TP63 binding to the SE regions of lncRNA LINC01503 led to LINC01503 overexpression in squamous cell cancer (18).

Many lncRNA databases and tools have been built. For example, NONCODE (19), LNCipedia (20), and RNAdb focus on providing basic annotation information for lncRNAs. LncRNADisease (21) and Lnc2Cancer (22) collect the details of relationships between lncRNAs and diseases. LncRNASNP (23), lnc2Meth (24) and LncVar (25) support lncRNAs interacting with other functional elements. StarBase (26) and LncBase (27) provide information on lncRNA targets. Such databases serve as valuable resources for the study of lncRNAs. However, they provide incomplete lists of lncRNAs, rather than a comprehensive, taxonomic set of lncRNAs for users. Moreover, those tools also lack information about the upstream transcriptional regulation of lncRNAs. With studies of human disease and biological processes, a large number of functional lncRNA sets have been generated from high-throughput or low-throughput experiments. The development of a comprehensive collection of human lncRNA sets is urgent work at present. Importantly, based on such reference lncRNA sets, enrichment analyses will be useful for analyzing lncRNA lists of interest submitted by users.

To infer lncRNA functions, some web servers and tools were developed, such as Co-LncRNA (28), Lnc-GFP (29) and FARNA (30); however, such tools analyze lncRNA functions using RNA-seq data, and the co-expression relationships between mRNAs and lncRNAs. Most tools fail to support enrichment analyses for a lncRNA set that provides only functional annotations for a single lncRNA. A web server, LnCompare (31), can be used to analyze lncRNA set features with six categories of >100 attributes. However, insufficient category characteristics may limit inferences of lncRNA function. Therefore, it is highly desirable to construct a comprehensive resource for lncRNA sets and provide lncRNA set annotation and enrichment analyses.

Here, we developed a human lncRNA sets database (LncSEA, http://bio.liclab.net/LncSEA/index.php), which focuses on accommodating various available resources of human lncRNAs and performs annotation and enrichment analyses of lncRNA lists submitted by users. LncSEA supports >40 000 reference lncRNA sets across 18 categories (miRNA, drug, disease, methylation pattern, cancer specific phenotype, lncRNA binding protein, cancer hallmark, subcellular localization, survival, lncRNA-eQTL, cell marker, enhancer, super-enhancer, transcription factor, accessible chromatin and smORF, exosome and conservation) and 66 sub-categories, which include over 50 000 lncRNAs. We collected lncRNA sets from >20 lncRNA-associated databases that generated lncRNA sets based on downstream regulatory data sources. Furthermore, by integrating TF ChIP-seq, DNase-seq, ATAC-seq and H3K27ac ChIP-seq data from hundreds of human cell types, we identified a large number of lncRNA sets regulated by upstream TFs and DNA regulatory elements. More importantly, LncSEA provides annotation and enrichment analyses of lncRNA set. Moreover, lncRNA set enrichment analyses associated with upstream regulators and downstream targets of lncRNAs can be performed simultaneously when choosing the categories for upstream and downstream reference sets. Finally, the differences and advantages of LncSEA compared to other existing databases or web tools, in terms of data and functionality, are shown in Supplementary Table S1 and Supplementary Material 1. In summary, LncSEA is a powerful platform that provides a variety of types of lncRNA sets for users, and performs annotation and enrichment analyses of lncRNA set submitted by users.

## DATA SOURCE AND PROCESSING

### Collection of reference sets of lncRNAs

**Figure 1.**
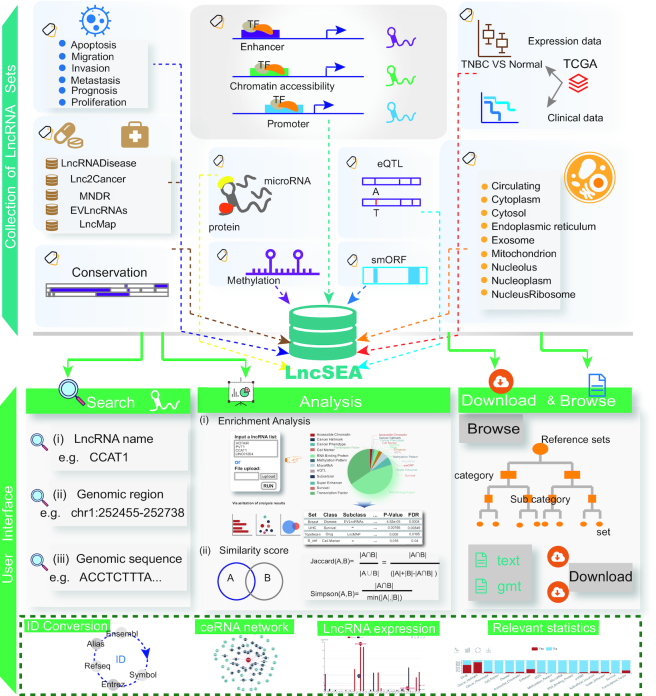
Collection of lncRNA sets and the user interface of LncSEA. LncSEA provides 18 types of reference sets, including miRNA, drug, disease, methylation pattern, cancer specific phenotype, lncRNA binding protein, cancer hallmark, subcellular localization, survival, eQTL, cell markers, enhancer & super enhancer, transcription factor, accessible chromatin, smORF, exosome and conservation. LncSEA supports multiple functions, including search, download, browse and enrichment analysis. ID conversion, ceRNA network, lncRNA expression and statistics are also provided in LncSEA.

LncSEA contains comprehensive collections of lncRNA sets (Figure 1). The current version of LncSEA contains >40 000 reference sets, including 18 categories and 66 sub-categories (Table 1). There are two types of sources for all of the reference sets, including sets identified by high-throughput experimental data and sets collected from >20 known databases. Seven of the 18 categories contain lncRNA sets from literature searches. For example, most of the lncRNAs in our reference sets of the ‘Disease’ and ‘Drug’ categories were confirmed by a large number of studies involving biological experiments. All of the lncRNA sets for the ‘Cell marker’, ‘Subcelluar localization’, ‘Cancer hallmark’ and ‘Exosome’ categories are composed of a list of lncRNAs selected purely manually in one or more studies.

**Table 1.** Overview of all the categories in LncSEA

No.	Category	Sub-category	Data resource	Number of sets
**1**	Accessible chromatin	Overlap; Proximal; Closest	Cistrome; NCBI; TCGA	1253
**2**	Enhancer	Overlap; Proximal; Closest	ENCODE; Roadmap; NCBI; GGR	253
**3**	Super enhancer	Overlap; Proximal; Closest; Closest active	ENCODE; Roadmap; NCBI; GGR	2150
**4**	Transcription factor	Enhancer; Accessible; Chromatin; Promoter	ENCODE; Remap; Cistrome; ChIP-Atlas; GTRD	14 478
**5**	Survival	/	TCGA	33
**6**	smORF	sorf.org; SmProt	sorf.org (32); SmProt (33)	15
**7**	Cancer hallmark	/	CRlncRNA (34)	7
**8**	Cancer phenotype	28 kinds of cancers such as Breast Carcinoma	Cancer RNA-Seq Nexus (35)	470
**9**	Cell marker	/	CellMarker	67
**10**	Disease	LncRNADisease; Lnc2Cancer; MNDR; EVLncRNAs	LncRNADisease2.0; Lnc2Cancer2.0; MNDR2.0 (36); EVLncRNAs (37)	1199
**11**	Drug	Lnc2Cancer; LncMap	Lnc2Cancer2.0; LncMap (38)	155
**12**	EQTL	Common cis; Common trans; GWAS cis; GWAS trans;	ncRNA-eQTL (39)	126
**13**	Methylation pattern	/	Lnc2Meth	5
**14**	MicroRNA	StarBase; LncBase	StarBase2.0; LncBase2.0	1231
**15**	RNA binding protein	RNAInter; EuRBPDB; StarBase	RNAInter (40); EuRBPDB (41); StarBase	18 717
**16**	Subcellular localization	RNALocate; iLoc-lncRNA	RNALocate (42); iLoc-lncRNA (43)	14
**17**	Exosome	/	exoRBase (44)	1
**18**	Conservation	100_vertebrates; 20_mammals; 7_vertebrates	LnComapre	18

LncRNAs are regulated by different regulatory elements and TFs, which bind to their regulatory regions. Due to data resource and technology constraints, few databases provide upstream regulatory information for lncRNAs. We constructed four categories of lncRNAs with upstream regulatory information involving ‘Enhancer’, ‘Super Enhancer’, ‘Accessible Chromatin’ and ‘Transcription Factor’ by collecting and processing large volumes of ChIP-seq/DNase-seq/ATAC-seq data (Table 1, No. 1–No. 4). Detailed descriptions of the reference collections for the different categories are as follows:

#### Enhancer and super enhancer categories

To build the ‘Enhancer’ and ‘Super Enhancer’ category sets (Table 1, No. 2 and No. 3), we collected and processed H3K27ac ChIP-seq data from NCBI GEO/SRA (45), ENCODE (46), Roadmap (47) and GGR (Genomics of Gene Regulation Project) (46) (The sample information of these datasets in Supplementary Table S2). To perform normalization and ensure consistency across different data sources, we used the streamlined pipeline of Bowtie-MACS-ROSE, which was developed by Loven *et al.* (48). Raw sequencing reads were aligned to the hg19 reference genome using Bowtie (49,50), peaks were called using MACS (51), and SE regions were annotated using ROSE (48) software. More than 330 000 SE regions from 542 cells/tissues were obtained. Detailed super enhancer annotation information and analyses were viewed in the SEdb (52) database and SEanalysis (53) web server developed by our team. Based on these enhancers and SEs, we identified the lncRNAs regulated by cell-type-specific enhancers and SEs using the ROSE software GeneMapper program (48). Three different positional relationships, including ‘overlap’, ‘proximal’ and ‘closest’ were supported between enhancers and lncRNAs. The enhancer-associated lncRNAs were classified into ‘overlap’ when the enhancer region overlapped by at least one base with the corresponding lncRNA. LncRNAs were classified into the ‘proximal’ sub-category when the distance between the enhancers and lncRNAs was within 50 kb, and they were classified into the ‘closest’ sub-category when the lncRNA was the closest gene and the distance was within 1000 kb. We constructed multiple sets for the closest active lncRNAs, with SEs identified by the CRC Mapper program (54) in specific cell types.

#### Accessible chromatin category

DNase-seq and ATAC-seq (46,55) technologies can be used to identify chromatin accessibility regions. (The sample information of these datasets in Supplementary Table S2). We collected the chromatin accessibility regions from DNase-seq data including 292 sample types from ENCODE, Roadmap and Cistrome (55). For ATAC-seq data, we collected the genomic regions of 105 sample types from Cistrome and NCBI, and 386 samples from 23 cancer types from TCGA (56) (https://tcga-data.nci.nih.gov/tcga). We used the liftOver tool in UCSC (57) to convert the genomic locations of those regions into hg19 version. The GeneMapper program in ROSE software (48) was also used to predict the chromatin accessibility regions associated with lncRNAs using the proximity rules, closest, overlapping, and proximal (Table 1, No. 1).

#### Transcription factor category

For the ‘Transcription Factor’ category (Table 1, No. 4), we collected TF ChIP-seq data for 467 sample types from ENCODE, Remap (58), Cistrome, ChIP-Atlas (http://chip-atlas.org) (59) and GTRD (60). The peaks overlapping with transcriptional regulatory regions were further identified using BEDTools (default parameter: at least one base overlapping) (61), including enhancers, promoters, and the chromatin accessibility regions of lncRNAs. Then, the relationships between TFs and lncRNAs were built via multiple kinds of lncRNA-related regulatory regions, such as promoter and enhancer regions bound by TFs. Finally, for each TF, we established lncRNA sets with cell/tissue-specific regulatory information.

#### Survival category

Some survival interacted lncRNAs were predicted by downloading and analyzing lncRNA expression data and clinical data. Univariate Cox regression analysis (62) was used to screen for lncRNAs related to prognosis. We defined each cancer survival related lncRNAs as a set in the TCGA project. Cox regression coefficients, *P*-values, and log rank test *P*-values are displayed on detailed set pages in our database for user screening and reference purposes. Our survival sets inform and guide the study of prognosis and lncRNA expression in cancer patients.

#### Other categories

We collected data for multiple categories of lncRNA sets surrounding human diseases and cancers, including cancer hallmarks, diseases, and drug target information from public databases (Table 1, No. 5–No. 14). Interactions between lncRNAs and downstream targets, and other supplementary information have been integrated in many public data sources such as StarBase, LncBase, and RNAInter (Table 1, No. 15 and No. 16). In addition, we collected subcellular localization information for lncRNAs and marker lncRNAs that define cell identity (Table 1, No. 9 and No. 11). To unify lncRNA names and eliminate duplicate lncRNAs, we converted all lncRNA names into official gene symbols. For those lncRNAs without official names, the original names were maintained. The detailed statistics and sample information of each dataset are provided in Supplementary Table S2. Detailed descriptions, methods of all reference collections, and software versions for data processing are provided in Supplementary Table S3 and Supplementary Material 2.

### Classification of all reference sets in LncSEA

We developed some rules for lncRNA classification, including: (i) directly classifying them into sub-categories based on the data source. For example, we included their relationships to disease from the Lnc2Cancer, LncRNADisease, MNDR, and EVLncRNAs databases. According to the first criterion, we divided the ‘Disease’ category into four sub-categories, and others such as ‘Drug’ in this way as well. (ii) Because several categories had special attributes and showed obvious root properties, we divided them into different sub-categories according to the characteristics of each category. For example, the ‘Cancer Phenotype’ category represented substantial collections of lncRNAs involving >30 different cancer types. We defined each cancer type as a subclass of this category. (iii) For the three categories of ‘Enhancer’, ‘Super Enhancer’ and ‘Accessible Chromatin’, we classified them into three sub-categories: ‘closest’, ‘overlap’ and ‘proximal’, which was based on the relationship between enhancers/super-enhancers/accessible chromatin and lncRNAs predicted by the ROSE program. (iv) We classified the ‘Transcription Factor’ category into six sub-categories, including four types of promoters with different distances to TSS, enhancer, and accessible chromatin. Based on the classification rules, we sorted all of the collected lncRNA sets (Table 1).

### Introduction of additional data sources

LncSEA provided additional information that helps users study lncRNA functions in depth. We obtained references for lncRNAs such as clinical information, biological function, and experimentally-supported mechanisms from the Lnc2Cancer2.0 database. The relationships between lncRNA, mRNA, and miRNA were obtained from an excellent ceRNA database, LncACTdb2.0 (63). Multiple lncRNA names, including gene symbol, Ensembl ID, NCBI refseq ID, alias, and Entrez ID were obtained from org.Hs.eg.db (Release 3.11). Chromatin location information for lncRNAs were download from GENCODE Hg19. Gene expression matrices with the FPKM value for invasive breast invasive carcinoma and prostate adenocarcinoma were obtained as test data from the TCGA project. The differentially expressed lncRNAs (*P*_adj_ < 0.05) of both cancers were obtained from the circlncRNAnet database. We downloaded lncRNA expression profiles with FPKM values from the TCGA project, and expression profiles with TPM values from GTEx, CCLE and ENCODE databases were normalized by log_2_(value + 1).

### LncRNA set enrichment and similarity analysis

LncRNA sets enrichment analyses can be performed on >40 000 reference sets, which are divided into 18 categories and 66 subcategories. The annotation and enrichment analyses based on these categories and reference sets in LncSEA covered >50 000 lncRNAs. Users can submit a list of lncRNAs and select multiple categories and various sub-categories of lncRNA sets according to their preferences. LncSEA will annotate lncRNAs submitted by users to the reference sets, and calculate the statistical significance of enrichment analyses using the hypergeometric test (64). The enrichment significance *P*-value for that reference set is calculated as:

(1) $$\begin{equation*}P = 1 - \mathop \sum \limits_{i\ = \ 0}^{x - 1} \frac{{\left( {\begin{array}{@{}*{1}{c}@{}} k\\ i \end{array}} \right)\left( {\begin{array}{@{}*{1}{c}@{}} {n - k}\\ {s - i} \end{array}} \right)}}{{\left( {\begin{array}{@{}*{1}{c}@{}} n\\ s \end{array}} \right)}}\ \end{equation*}$$

We consider that reference sets have a total of n lncRNAs (LncSEA or GENCODE), of which k are components of one reference set under investigation, and the query list of lncRNAs of interest has a total of s lncRNAs, of which i are involved in the same reference set. Thus, the enrichment significance *P*-value for that reference set is calculated using formula (i). Users can adjust the number of lncRNAs required to be enriched, and set thresholds for *P*-values, false discovery rates (FDR), and the Bonferroni method to control the accuracy of the analysis.

To evaluate the similarity between a query lncRNA set, A, and a reference lncRNA set, B, we applied two classical measures for computing set similarity. The first one was the Jaccard score, which represents a proportion of the intersection elements of the two sets, A and B, in the union set of A and B. The second measure was the Simpson score, which represents a proportion of intersection elements of the two sets, A and B, in the minimum set of A and B.

The Jaccard score was calculated as:

(2) $$\begin{equation*}{\rm{Jaccard }}\left( {{\rm{A}},{\rm{B}}} \right) = \frac{{\left| {{\rm{A}} \cap {\rm{B}}} \right|}}{{\left| {{\rm{A}} \cup {\rm{B}}} \right|}}{\rm{\ }} = \frac{{\left| {{\rm{A}} \cap {\rm{B}}} \right|}}{{\left| {\rm{A}} \right| + \left| {\rm{B}} \right| - \left| {{\rm{A}} \cap {\rm{B}}} \right|}}\end{equation*}$$

The Simpson score was calculated as

(3) $$\begin{equation*}{\rm{Simpson }}\left( {{\rm{A}},{\rm{B}}} \right) = \frac{{\left| {{\rm{A}} \cap {\rm{B}}} \right|}}{{{\rm{min}}\left( {\left| {{\rm{A}}\left| , \right|{\rm{B}}} \right|} \right)\ }}\end{equation*}$$

These two scores can provide additional information for enrichment analyses, and also allow users the choice of more parameters to deepen their understanding of the analytical results.

### Similarity calculation between reference lncRNA sets

To provide users with a deep understanding of the reference lncRNA sets, we provided an analysis module to calculate the similarity between reference sets. The similarity score between any two sets in the whole reference set can be quickly computed by the two measures (Formulae (2), (3)) in our database. Users can discover potential associations between two reference sets across the same or different category by browsing the details of each set. Users can not only find directly-related sets by querying a lncRNA list of interest, but can also calculate similarities to other sets to identify indirectly-related sets for lncRNAs. In addition, users can identify relationships between two categories by calculating the similarity scores between sets, which will contribute to the exploration and study the unknown lncRNA functions.

## SYSTEM DESIGN AND IMPLEMENTATION

The current version of LncSEA was organized using MySQL 5.7.17 (http://www.mysql.com) and operates on a Linux-based Aliyun Web server. The website was developed based on PHP5.4.45.0 (http://www.php.net), CSS3, and HTML5 frameworks. The lncSEA web interface was designed and built using Bootstrap v3.3.7 (https://v3.bootcss.com) and JQuery v2.1.1 (http://jquery.com). Additionally, we used server-side R scripts for lncRNA set enrichment analysis. Our platform is convenient for users to access and use as it does not require users to register or login to access the database. We recommend using a modern web browser that supports HTML5, such as Firefox and Google Chrome for the best display. The LncSEA database is freely available to the research community at the following web address (http://bio.liclab.net/LncSEA/index.php). R script of enrichment analysis and PHP program are provided in Github website. (https://github.com/lxy-boy/LncSEA-Code)

## DATABASE USE AND ACCESS

### Overview of LncSEA database

The main elements of LncSEA, including the collection of lncRNA sets and the user interface are shown in Figure 1. The current version of LncSEA contains >40 000 reference sets across 18 categories and 66 sub-categories. ‘Transcription factor’-, ‘lncRNA binding protein’-, and ‘super-enhancer’-related collections were among the top three in total number (Figure 1). LncSEA provides a user-friendly interface to query, browse, and download detailed information about all of the reference lncRNA sets (Figure 1). In particular, LncSEA provides enrichment analyses of lncRNA sets.

### Effective online tool for lncRNA set enrichment analysis

**Figure 2.**
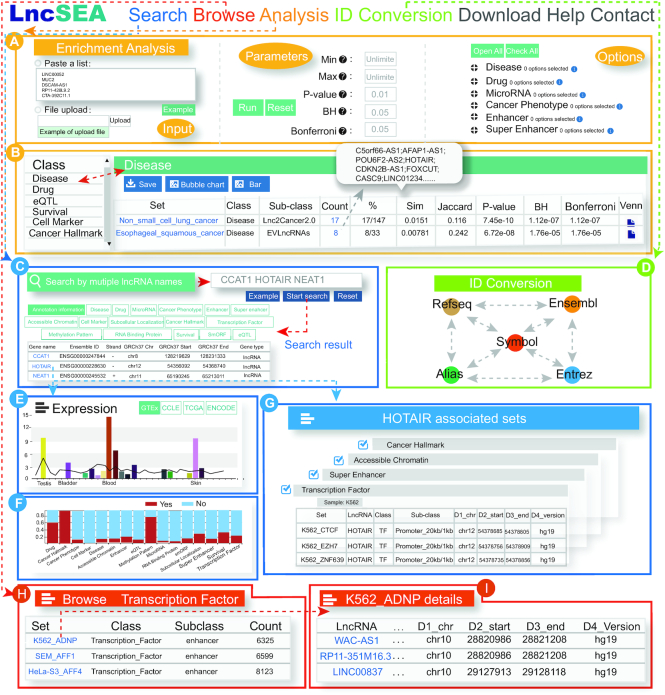
Introduction and usage of LncSEA. (**A**) Preparation of enrichment analyses, including input, parameters, and options. (**B**) Results table of enrichment analysis. (**C**) Users can search lncRNA using three methods, including ‘Search by lncRNA name’, ‘Search by genomic region’ and ‘Search by genomic sequence’. (**D**) ID conversion. (**E**) Bar plot of lncRNA HOTAIR expression in different samples. (**F**) Stacked graphs show the number of lncRNA-related sets in different categories. The red portion represents the set proportion annotated with lncRNAs, and the blue portion represents the set proportion not annotated. (**G**) Sets associated with the query lncRNA, HOTAIR. (**H, I**) Browse and details of lncRNA sets.

LncSEA provides lncRNA set enrichment analyses for users. The lncRNA set enrichment analyses that are associated with upstream regulators and downstream targets of lncRNAs can be performed simultaneously. To perform the enrichment analysis, users must input an lncRNA list of interest or a text file containing lncRNAs of interest and select the categories and sub-categories of the reference sets, as well as the parameters and background sets (LncSEA or GENCODE) (Figure 2A). Then, LncSEA will annotate lncRNAs to the reference lncRNA sets, and calculate the statistical significance of the enrichment and similarity scores using the hypergeometric test. Once running, the site will display a progress bar as a percentage to estimate the analytical time. All of the relevant collection categories are shown on the left panel of the return page, and users can view each category according to their own interests. The right panel displays the enrichment analysis results. Users can select the top blue buttons to download the results tables, plot the enrichment analysis bubble and generate a bar chart. Each column of the table represents the name of the lncRNA set, category, sub-category, number of annotated lncRNAs, proportion, Jaccard score, Simpson score, enrichment analysis *P*-value and adjusted *P*-value. Users can click on the ‘set’ hyperlink to view the set details, as well as similar sets. Users can also obtain lncRNA names annotated to the set by clicking the ‘count’ hyperlink. All significant reference lncRNA sets and visualization results for the enrichment analysis are provided for review and download (Figure 2B).

### Search interface for conveniently retrieving lncRNA sets

Users can search for lncRNAs and their related categories using three approaches: (i) multiple lncRNA features, including gene symbol, Ensembl ID, NCBI refseq ID, alias, and Entrez ID, (ii) genomic region and (iii) genomic sequence (Figure 2C). If users search via a genome sequence, the sequence alignment from the basic local alignment search tool (BLAST) (65) is also available to download. The results page of the query returns the lncRNA basic information, including gene symbol, Ensembl ID, genomic region, and lncRNA type. The results page also returns all sets related to the query lncRNAs and users can view them by clicking each category. If users only wish to browse one lncRNA in detail, they can select the lncRNA name using the hyperlink. The detailed information associated with lncRNAs will be displayed, such as references to the lncRNA, the sets associated with the lncRNA, sets statistics, and lncRNA expression in different samples of GTEx projects (66), TCGA projects (https://tcga-data.nci.nih.gov/tcga) normal and cancer, ENCODE projects, and CCLE (67) projects (Figure 2E and F). Users can view references for each lncRNA, which are including clinical information, biological function, and experimentally-supported mechanism to rapidly understand the related functions of lncRNAs. For a lncRNA-associated set, users can obtain set names associated with the lncRNA, the category and subcategory to which the set belongs, and lncRNA number of the set by selecting the category. All relevant collections and evidence of current lncRNAs are presented in different modules according to the category on the results page. For example, the ‘Transcription_Factor’ module shows the specific genomic regions for which three transcription factors, CTCF, EZH2, and ZNF639 regulated the promoter regions of lncRNA HOTAIR in K562 cells. Users can select the set hyperlink to review the set details and select samples from the drop-down menu above the table to view TF regulatory information (Figure 2G). To facilitate further study of the function and mechanism of lncRNAs, lncRNA-associated ceRNA networks are also displayed in the module at the bottom of the results page. LncSEA provided two types of networks based on experimental validations and predictions based on TCGA cancer datasets. Three different types of nodes, including lncRNA, miRNA, and protein coding mRNA in the network are represented by three colors. Users can also drag the edges and nodes to adjust the layout of the ceRNA network. Additionally, users can download images and tables for the ceRNA network (Figure 2H).

### User-friendly interface for browsing lncRNA sets

The ‘Browse’ page is organized as an interactive table that allows users to quickly search for lncRNA sets and customize filters according to ‘Class’ and ‘Sub class’. Users can click the ‘Show entries’ drop-down menu to change the number of displayed records per page. To view the details of a given lncRNA set, users only need to click on the ‘Set’ option. The details of the selected lncRNA set include the categories to which the set belongs, the list of lncRNA names in the set, and the evidence supporting the relationships between the set and each lncRNA. For example, when users select the ‘Transcription Factor’ class set and the ‘enhancer’ sub-class, the right side of the interface will show the corresponding set. Each column in the table shown on the right side represents the set name, the class attached to the set, the subclass attached to the set, and the number of lncRNAs contained in the set (Figure 2H and I).

### ID conversion

LncSEA also supports a user-friendly ‘ID conversion’ function (Figure 2D). Users can paste an lncRNA list or upload a file separated by spaces with multiple lncRNA names, including gene symbol, Ensembl ID, NCBI refseq ID, alias, and Entrez ID. When selecting the ‘Convert’ option, users can obtain the converted results table. Users can not only download the results table, but can also check the ‘Analysis’ option to connect to the enrichment analysis page for those lncRNAs.

### Data download

The ‘Download’ page was organized as an interactive table. All reference sets of lncRNAs have been arranged and sorted into separate files for download in our database. We also provide two types of file formats for download, including .gmt and .txt. Users can download the reference collection as valuable supplementary data for in-depth experimental research.

### A case study using differential cancer lncRNAs

**Figure 3.**
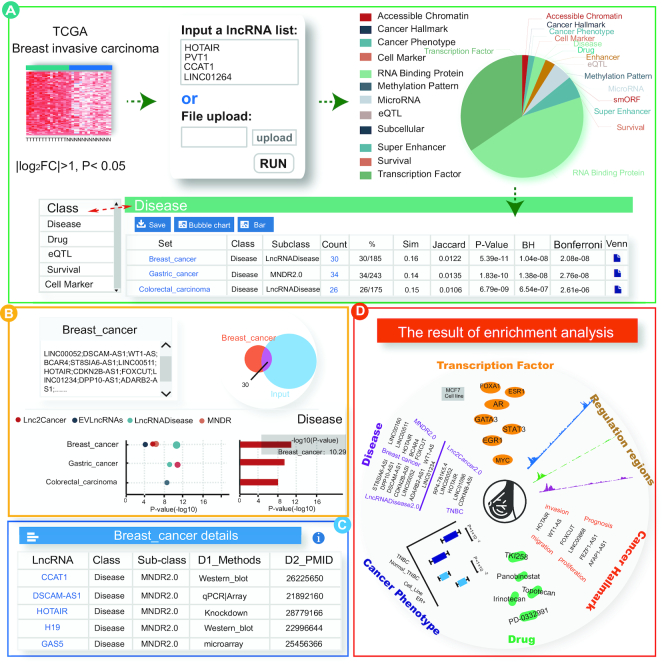
Results of enrichment analyses associated with 1000 up-regulated lncRNAs in invasive breast invasive cancer. (**A**) Results table for enrichment analyses of the ‘Disease’ category. (**B**) Validation results for enrichment analysis of the ‘Disease’ category. (**C**) Detailed information table of the ‘Breast_cancer’ sets. (**D**) The results of enrichment analysis.

To find lncRNAs as therapeutic and drug targets for breast cancer, numerous studies have focused on identifying differentially expressed lncRNAs. To further explore the function of differential lncRNAs, enrichment analyses of such lncRNAs is necessary. Thus, we used LncSEA to perform functional analyses on the genes differentially expressed in breast cancer. Firstly, we obtained those lncRNAs (log_2_FC > 1, *P*_adj_ < 0.05) of breast invasive cancer from the TCGA project and circlncRNAnet (68) database as inputs for LncSEA. Next, we set the parameters to include the hypergeometric test *P*-value = 0.01 and adjusted the *P*-value = 0.05, and selected the ‘RUN’ button to perform the enrichment analysis. A total of 18 categories for the sets, including ‘transcription factors’, ‘Disease’, ‘Drug’, ‘Enhancer’, ‘eQTL’ and ‘Cancer_Phenotype’ were returned on the left panel of the interface (Figure 3A). The detailed gene annotation and enrichment analysis results are shown in Supplementary Table S4.

We found from the analytical results that these differential lncRNAs were closely related to cancer and therapeutic drugs. For example, when clicking on the ‘Disease’ set class, we found that the lncRNAs were significantly enriched to two ‘Breast_cancer’ reference sets that belonged to the MNDR2.0, Lnc2Cancer2.0, EVLncRNAs and LncRNADisease2.0 sub-classes. There were 39 lncRNAs annotated to the breast cancer sets, such as the star molecule HOTAIR, which was reported as a cancer biomarker and therapeutic target (69), and a tumor-suppressor DNA boundary element (70) (Supplementary Table S4). The bubble, bar graphs and Venn diagram of the enrichment analysis results were also provided (Figure 3B). By selecting ‘Breast_cancer’, all of the evidence for correlations between lncRNAs and ‘Breast_cancer’ were listed in the tables on each page. For example, lncRNA HOTAIR was proven by qPCR and knockdown experiments (71) to be associated with breast cancer (Figure 3C). To further study the subtypes of cancer, LncSEA provided the function of lncRNA enrichment analysis for cancer phenotypes. We found that most of the lncRNAs were significantly enriched to the ‘Breast_cancer_ER + VS Breast_cancer_Normal_TNBC’ set (Simpson score = 0.589, *P* < 0.01) and the ‘Breast_cancer_TNBC VS Breast_cancer_Normal_TNBC’ set (*P* < 0.01, Simpson score = 0.541) (Supplementary Table S4). Researchers can studied the classification of breast cancers by comparing the biological functions of these two phenotype-specific lncRNAs.

The identification of novel drug targets and the development of new candidate drugs are of great significance for the targeted treatment of cancer. In the results section of the ‘Drug’ category, we found these lncRNAs were significantly enriched to the anti-breast cancer drugs Topotecan and Panobinostat (72,73). Interestingly, the drug, TKI258, that ranked third most significant in the enrichment analysis was recently reported to lead to the suppression of downstream signaling by RAS-RAF-MAPK and PI3K-AKT molecules, which are involved in cell proliferation, cell survival, and tumor invasion (74). This result suggested that some of the up-regulated lncRNAs in cancer samples might be used as TKI258 targets. Some studies showed that mutations in lncRNAs may lead to changes in lncRNA expression levels. In the ‘eQTL’ category, the results showed that the differentially expressed lncRNAs were significantly enriched in breast cancer samples (*P* < 0.01, Simpson score = 0.337).

Although the analyses above showed that differentially-expressed genes were highly related to breast cancer, further explanation of the biological mechanisms leading to cancer is even more important. We speculated that most differentially expressed lncRNAs were regulated by upstream transcriptional regulators, which affected expression levels and lead to breast cancer. It is worth noting that some of the results are consistent with our hypothesis (Figure 3D). The enrichment results showed that most of the lncRNAs were regulated by accessible chromatin, enhancers, and SE regions in breast cancer tissues and samples. The details of the regulatory regions and more details are available at the LncSEA website (Supplementary Table S4). In addition, these genes are significantly enriched in some core transcription factors in the MCF-7 cell line, such as FOXA1, ESR1, and GATA3, which are important players in transcriptional regulatory networks in breast cancer (75). Several novel TFs with high enrichment scores such as KDM5B have not been widely studied in breast cancer, suggesting that they might be potential novel genes associated with breast cancer. The results of enrichment analyses for more categories can be observed in Supplementary Table S4.

To verify the function and accuracy of LncSEA, we compared the differences between the differentially expressed group and the other three groups included in the enrichment analyses. To maintain consistency across all independent variables, we selected the same scale sets with differentially expressed lncRNAs (log_2_FC > 1; *P* < 0.05; 2306 lncRNAs) from the breast cancer expression profile. First, we sorted the lncRNAs according to the average expression in all samples. Then, we used high expression, low expression and random 100 times group test sets as inputs for the enrichment analyses. By counting the number of enrichment categories and sets, we found that the four groups had significantly different enrichment results (Supplementary Figure S1). The enrichment results for the high expression group and differentially expressed group were similar. However, the differently expressed group was enriched with more upstream regulatory elements and factor sets than the high expression group. This result indicated that some lncRNAs were specifically and highly expressed in cancer as they were regulated by upstream regulatory elements and factors during the transcription process, and those lncRNAs were more likely to be drug targets. Consistent with our expectations, the high expression group was more related to disease, cancer phenotype and RNA binding protein. In contrast, the low expression and random groups were both enriched to a few categories and sets not related to breast cancer (Supplementary Figure S1). We also performed the same tests for prostate cancer and obtained similar results (Supplementary Figure S1). Collectively, we provided a specific case study with enrichment analyses and random tests in two different cancers. The results demonstrate the availability and biological value of LncSEA in lncRNA research.

## DISCUSSION

The emerging importance of lncRNAs in human diseases and biological processes, coupled with their upstream regulators and downstream target genes, increases the need for comprehensive human lncRNA reference sets. Therefore, we constructed a human lncRNA database, called LncSEA. Compared with all existing lncRNA databases, LncSEA focuses on building comprehensive human lncRNA sets, and has collected the largest number of human lncRNA sets to date (Supplementary Table S1). LncSEA supports >40 000 reference lncRNA sets, and over 50 000 lncRNAs are annotated to at least one lncRNA set in LncSEA. Thus, such lncRNA sets not only included the sets associated with downstream regulatory data, but also a large number of sets regulated by upstream TFs and DNA regulatory elements by integrating TF ChIP-seq, DNase-seq, ATAC-seq, and H3K27ac ChIP-seq data. Importantly, based on those reference sets, LncSEA provides lncRNA set annotation and enrichment analysis. Although many databases and tools, such as DAVID (76), GSEA (77), MIEAA (78), TAM2.0 (79) and ESEA (80) provide enrichment analysis for gene set, they mainly focus on the analysis of coding gene, miRNA and pathway, rather than lncRNA set. LncSEA provides annotation and enrichment analysis on lncRNA set, as well as their associated upstream regulators and downstream targets.

LncSEA supports a user-friendly interface to analyze, query, browse and download detailed information on lncRNA sets. The main advantages of the database are illustrated below: (I) LncSEA provides comprehensive lncRNA reference sets with classifications of lncRNA sets. There are >40 000 reference lncRNA sets classified into 18 categories and 66 sub-categories. (II) LncSEA supports enrichment analyses for lncRNA sets of interest. In particular, users can perform enrichment analyses of lncRNAs of interest associated with upstream regulators and downstream targets to infer their functions. (III) LncSEA supports the visualization and download of enrichment analysis results. (IV) Users can quickly search related sets by using different lncRNA names; (V) users can quickly search related sets based on genomic region or sequence; and (VI) users can browse each reference lncRNA set. LncSEA provides a catalogue, including categories and sub-categories to browse lncRNA sets. (VII) Similarity score analyses between any two reference lncRNA sets can also be provided by LncSEA. (VIII) ID conversion function is also provided by LncSEA and (IX) LncSEA supports user-friendly displays and allows the download of reference lncRNA sets with interactive tables.

Our effort to establish this platform was prompted by the need of researchers to perform functional analyses of lncRNA sets. Such researchers include geneticists, cell/molecular biologists, and bioinformaticians. Moreover, the field of lncRNA is progressing faster than ever, and the enrichment analysis of a lncRNA set is an indispensable research strategy. LncSEA is a comprehensive resource for human lncRNA sets and is an analysis platform to enhance our understanding of lncRNA functions. The current version of LncSEA stores the most abundant human lncRNA sets and we will manually curate additional lncRNA sets in the future. There are some excellent algorithms and software (81,82) based network for predicting the relationships between lncRNAs and pathways, but because of the complexity of such relationships, we considered adding such data in the next version of LncSEA. Continuous efforts will be made to update the platform with the available data and improve the functionality of the LncSEA database.

## CONCLUSIONS AND EXPECTATIONS

The current version of LncSEA involved 18 categories, including >40 000 human lncRNA references. LncSEA is the first database providing a comprehensive collection of lncRNAs and is capable of performing enrichment analyses upstream and downstream of lncRNAs. With the development of new technologies and the accumulation of experimental data, an increasing number of lncRNA-related information will be generated. In the future, LncSEA will supplement more categories of lncRNAs and additional functional information by tracking developments in biology. We will also include additional experimental sets to extend our data source, and support more powerful enrichment analysis tool. In addition, we will strive to expand the number of species and collections, and provide users with more efficient enrichment analysis methods in the next version of LncSEA.

## Supplementary Material

gkaa806_Supplemental_Files

## References

[1] Huo X., Han S., Wu G., Latchoumanin O., Zhou G., Hebbard L., George J., Qiao L. Dysregulated long noncoding RNAs (lncRNAs) in hepatocellular carcinoma: implications for tumorigenesis, disease progression, and liver cancer stem cells. Mol. Cancer. 2017; 16:165. 29061150 10.1186/s12943-017-0734-4 PMC5651571

[2] Guttman M., Amit I., Garber M., French C., Lin M.F., Feldser D., Huarte M., Zuk O., Carey B.W., Cassady J.P. et al. Chromatin signature reveals over a thousand highly conserved large non-coding RNAs in mammals. Nature. 2009; 458:223–227. 19182780 10.1038/nature07672 PMC2754849

[3] Slack F.J., Chinnaiyan A.M. The role of non-coding RNAs in oncology. Cell. 2019; 179:1033–1055. 31730848 10.1016/j.cell.2019.10.017 PMC7347159

[4] Iyer M.K., Niknafs Y.S., Malik R., Singhal U., Sahu A., Hosono Y., Barrette T.R., Prensner J.R., Evans J.R., Zhao S. et al. The landscape of long noncoding RNAs in the human transcriptome. Nat. Genet. 2015; 47:199–208. 25599403 10.1038/ng.3192 PMC4417758

[5] Borrello M.G., Pierotti M.A., Tamborini E., Biassoni D., Rizzetti M.G., Pilotti S., Della Porta G. DNA methylation of coding and non-coding regions of the human H-RAS gene in normal and tumor tissues. Oncogene. 1992; 7:269–275. 1549348

[6] Li D., Da L., Tang H., Li T., Zhao M. CpG methylation plays a vital role in determining tissue- and cell-specific expression of the human cell-death-inducing DFF45-like effector A gene through the regulation of Sp1/Sp3 binding. Nucleic Acids Res. 2008; 36:330–341. 18033804 10.1093/nar/gkm1028 PMC2248752

[7] Oliva J., French B.A., Qing X., French S.W. The identification of stem cells in human liver diseases and hepatocellular carcinoma. Exp. Mol. Pathol. 2010; 88:331–340. 20080086 10.1016/j.yexmp.2010.01.003 PMC3593713

[8] Zhang X., Lan Y., Xu J., Quan F., Zhao E., Deng C., Luo T., Xu L., Liao G., Yan M. et al. CellMarker: a manually curated resource of cell markers in human and mouse. Nucleic Acids Res. 2019; 47:D721–D728. 30289549 10.1093/nar/gky900 PMC6323899

[9] Liu R., Li X., Zhu W., Wang Y., Zhao D., Wang X., Gurley E.C., Liang G., Chen W., Lai G. et al. Cholangiocyte-derived exosomal long noncoding RNA H19 promotes hepatic stellate cell activation and cholestatic liver fibrosis. Hepatology. 2019; 70:1317–1335. 30985008 10.1002/hep.30662 PMC6783323

[10] Ulitsky I., Bartel D.P. lincRNAs: genomics, evolution, and mechanisms. Cell. 2013; 154:26–46. 23827673 10.1016/j.cell.2013.06.020 PMC3924787

[11] Li W., Zhang Z., Liu X., Cheng X., Zhang Y., Han X., Zhang Y., Liu S., Yang J., Xu B. et al. The FOXN3-NEAT1-SIN3A repressor complex promotes progression of hormonally responsive breast cancer. J. Clin. Invest. 2017; 127:3421–3440. 28805661 10.1172/JCI94233 PMC5669564

[12] Matsumoto A., Pasut A., Matsumoto M., Yamashita R., Fung J., Monteleone E., Saghatelian A., Nakayama K.I., Clohessy J.G., Pandolfi P.P. mTORC1 and muscle regeneration are regulated by the LINC00961-encoded SPAR polypeptide. Nature. 2017; 541:228–232. 28024296 10.1038/nature21034

[13] Anderson D.M., Anderson K.M., Chang C.L., Makarewich C.A., Nelson B.R., McAnally J.R., Kasaragod P., Shelton J.M., Liou J., Bassel-Duby R. et al. A micropeptide encoded by a putative long noncoding RNA regulates muscle performance. Cell. 2015; 160:595–606. 25640239 10.1016/j.cell.2015.01.009 PMC4356254

[14] Xiang J.F., Yin Q.F., Chen T., Zhang Y., Zhang X.O., Wu Z., Zhang S., Wang H.B., Ge J., Lu X. et al. Human colorectal cancer-specific CCAT1-L lncRNA regulates long-range chromatin interactions at the MYC locus. Cell Res. 2014; 24:513–531. 24662484 10.1038/cr.2014.35 PMC4011346

[15] Qin Q., Fan J., Zheng R., Wan C., Mei S., Wu Q., Sun H., Brown M., Zhang J., Meyer C.A. et al. Lisa: inferring transcriptional regulators through integrative modeling of public chromatin accessibility and ChIP-seq data. Genome Biol. 2020; 21:32. 32033573 10.1186/s13059-020-1934-6 PMC7007693

[16] Peng L., Jiang B., Yuan X., Qiu Y., Peng J., Huang Y., Zhang C., Zhang Y., Lin Z., Li J. et al. Super-enhancer-associated long noncoding RNA HCCL5 is activated by ZEB1 and promotes the malignancy of hepatocellular carcinoma. Cancer Res. 2019; 79:572–584. 30482773 10.1158/0008-5472.CAN-18-0367

[17] Jiang Y., Jiang Y.Y., Xie J.J., Mayakonda A., Hazawa M., Chen L., Xiao J.F., Li C.Q., Huang M.L., Ding L.W. et al. Co-activation of super-enhancer-driven CCAT1 by TP63 and SOX2 promotes squamous cancer progression. Nat. Commun. 2018; 9:3619. 30190462 10.1038/s41467-018-06081-9 PMC6127298

[18] Xie J.J., Jiang Y.Y., Jiang Y., Li C.Q., Lim M.C., An O., Mayakonda A., Ding L.W., Long L., Sun C. et al. Super-enhancer-driven long non-coding RNA LINC01503, regulated by TP63, is over-expressed and oncogenic in squamous cell carcinoma. Gastroenterology. 2018; 154:2137–2151. 29454790 10.1053/j.gastro.2018.02.018

[19] Fang S., Zhang L., Guo J., Niu Y., Wu Y., Li H., Zhao L., Li X., Teng X., Sun X. et al. NONCODEV5: a comprehensive annotation database for long non-coding RNAs. Nucleic Acids Res. 2018; 46:D308–D314. 29140524 10.1093/nar/gkx1107 PMC5753287

[20] Volders P.J., Anckaert J., Verheggen K., Nuytens J., Martens L., Mestdagh P., Vandesompele J. LNCipedia 5: towards a reference set of human long non-coding RNAs. Nucleic Acids Res. 2019; 47:D135–D139. 30371849 10.1093/nar/gky1031 PMC6323963

[21] Bao Z., Yang Z., Huang Z., Zhou Y., Cui Q., Dong D. LncRNADisease 2.0: an updated database of long non-coding RNA-associated diseases. Nucleic Acids Res. 2019; 47:D1034–D1037. 30285109 10.1093/nar/gky905 PMC6324086

[22] Gao Y., Wang P., Wang Y., Ma X., Zhi H., Zhou D., Li X., Fang Y., Shen W., Xu Y. et al. Lnc2Cancer v2.0: updated database of experimentally supported long non-coding RNAs in human cancers. Nucleic Acids Res. 2019; 47:D1028–D1033. 30407549 10.1093/nar/gky1096 PMC6324001

[23] Miao Y.R., Liu W., Zhang Q., Guo A.Y. lncRNASNP2: an updated database of functional SNPs and mutations in human and mouse lncRNAs. Nucleic Acids Res. 2018; 46:D276–D280. 29077939 10.1093/nar/gkx1004 PMC5753387

[24] Zhi H., Li X., Wang P., Gao Y., Gao B., Zhou D., Zhang Y., Guo M., Yue M., Shen W. et al. Lnc2Meth: a manually curated database of regulatory relationships between long non-coding RNAs and DNA methylation associated with human disease. Nucleic Acids Res. 2018; 46:D133–D138. 29069510 10.1093/nar/gkx985 PMC5753220

[25] Chen X., Hao Y., Cui Y., Fan Z., He S., Luo J., Chen R. LncVar: a database of genetic variation associated with long non-coding genes. Bioinformatics. 2017; 33:112–118. 27605101 10.1093/bioinformatics/btw581

[26] Li J.H., Liu S., Zhou H., Qu L.H., Yang J.H. starBase v2.0: decoding miRNA-ceRNA, miRNA-ncRNA and protein-RNA interaction networks from large-scale CLIP-Seq data. Nucleic Acids Res. 2014; 42:D92–D97. 24297251 10.1093/nar/gkt1248 PMC3964941

[27] Paraskevopoulou M.D., Vlachos I.S., Karagkouni D., Georgakilas G., Kanellos I., Vergoulis T., Zagganas K., Tsanakas P., Floros E., Dalamagas T. et al. DIANA-LncBase v2: indexing microRNA targets on non-coding transcripts. Nucleic Acids Res. 2016; 44:D231–D238. 26612864 10.1093/nar/gkv1270 PMC4702897

[28] Zhao Z., Bai J., Wu A., Wang Y., Zhang J., Wang Z., Li Y., Xu J., Li X. Co-LncRNA: investigating the lncRNA combinatorial effects in GO annotations and KEGG pathways based on human RNA-Seq data. Database (Oxford). 2015; 2015:bav082. 26363020 10.1093/database/bav082 PMC4565967

[29] Guo X., Gao L., Liao Q., Xiao H., Ma X., Yang X., Luo H., Zhao G., Bu D., Jiao F. et al. Long non-coding RNAs function annotation: a global prediction method based on bi-colored networks. Nucleic Acids Res. 2013; 41:e35. 23132350 10.1093/nar/gks967 PMC3554231

[30] Alam T., Uludag M., Essack M., Salhi A., Ashoor H., Hanks J.B., Kapfer C., Mineta K., Gojobori T., Bajic V.B. FARNA: knowledgebase of inferred functions of non-coding RNA transcripts. Nucleic Acids Res. 2017; 45:2838–2848. 27924038 10.1093/nar/gkw973 PMC5389649

[31] Carlevaro-Fita J., Liu L., Zhou Y., Zhang S., Chouvardas P., Johnson R., Li J. LnCompare: gene set feature analysis for human long non-coding RNAs. Nucleic Acids Res. 2019; 47:W523–W529. 31147707 10.1093/nar/gkz410 PMC6602513

[32] Olexiouk V., Van Criekinge W., Menschaert G. An update on sORFs.org: a repository of small ORFs identified by ribosome profiling. Nucleic Acids Res. 2018; 46:D497–D502. 29140531 10.1093/nar/gkx1130 PMC5753181

[33] Hao Y., Zhang L., Niu Y., Cai T., Luo J., He S., Zhang B., Zhang D., Qin Y., Yang F. et al. SmProt: a database of small proteins encoded by annotated coding and non-coding RNA loci. Brief. Bioinform. 2018; 19:636–643. 28137767 10.1093/bib/bbx005

[34] Wang J., Zhang X., Chen W., Li J., Liu C. CRlncRNA: a manually curated database of cancer-related long non-coding RNAs with experimental proof of functions on clinicopathological and molecular features. BMC Med. Genet. 2018; 11:114. 10.1186/s12920-018-0430-2 PMC6311896 30598113

[35] Li J.R., Sun C.H., Li W., Chao R.F., Huang C.C., Zhou X.J., Liu C.C. Cancer RNA-Seq Nexus: a database of phenotype-specific transcriptome profiling in cancer cells. Nucleic Acids Res. 2016; 44:D944–D951. 26602695 10.1093/nar/gkv1282 PMC4702907

[36] Cui T., Zhang L., Huang Y., Yi Y., Tan P., Zhao Y., Hu Y., Xu L., Li E., Wang D. MNDR v2.0: an updated resource of ncRNA-disease associations in mammals. Nucleic Acids Res. 2018; 46:D371–D374. 29106639 10.1093/nar/gkx1025 PMC5753235

[37] Zhou B., Zhao H., Yu J., Guo C., Dou X., Song F., Hu G., Cao Z., Qu Y., Yang Y. et al. EVLncRNAs: a manually curated database for long non-coding RNAs validated by low-throughput experiments. Nucleic Acids Res. 2018; 46:D100–D105. 28985416 10.1093/nar/gkx677 PMC5753334

[38] Li Y., Li L., Wang Z., Pan T., Sahni N., Jin X., Wang G., Li J., Zheng X., Zhang Y. et al. LncMAP: Pan-cancer atlas of long noncoding RNA-mediated transcriptional network perturbations. Nucleic Acids Res. 2018; 46:1113–1123. 29325141 10.1093/nar/gkx1311 PMC5815097

[39] Li J., Xue Y., Amin M.T., Yang Y., Yang J., Zhang W., Yang W., Niu X., Zhang H.Y., Gong J. ncRNA-eQTL: a database to systematically evaluate the effects of SNPs on non-coding RNA expression across cancer types. Nucleic Acids Res. 2020; 48:D956–D963. 31410488 10.1093/nar/gkz711 PMC6943077

[40] Lin Y., Liu T., Cui T., Wang Z., Zhang Y., Tan P., Huang Y., Yu J., Wang D. RNAInter in 2020: RNA interactome repository with increased coverage and annotation. Nucleic Acids Res. 2020; 48:D189–D197. 31906603 10.1093/nar/gkz804 PMC6943043

[41] Liao J.Y., Yang B., Zhang Y.C., Wang X.J., Ye Y., Peng J.W., Yang Z.Z., He J.H., Zhang Y., Hu K. et al. EuRBPDB: a comprehensive resource for annotation, functional and oncological investigation of eukaryotic RNA binding proteins (RBPs). Nucleic Acids Res. 2020; 48:D307–D313. 31598693 10.1093/nar/gkz823 PMC6943034

[42] Zhang T., Tan P., Wang L., Jin N., Li Y., Zhang L., Yang H., Hu Z., Zhang L., Hu C. et al. RNALocate: a resource for RNA subcellular localizations. Nucleic Acids Res. 2017; 45:D135–D138. 27543076 10.1093/nar/gkw728 PMC5210605

[43] Su Z.D., Huang Y., Zhang Z.Y., Zhao Y.W., Wang D., Chen W., Chou K.C., Lin H. iLoc-lncRNA: predict the subcellular location of lncRNAs by incorporating octamer composition into general PseKNC. Bioinformatics. 2018; 34:4196–4204. 29931187 10.1093/bioinformatics/bty508

[44] Li S., Li Y., Chen B., Zhao J., Yu S., Tang Y., Zheng Q., Li Y., Wang P., He X. et al. exoRBase: a database of circRNA, lncRNA and mRNA in human blood exosomes. Nucleic Acids Res. 2018; 46:D106–D112. 30053265 10.1093/nar/gkx891 PMC5753357

[45] Barrett T., Troup D.B., Wilhite S.E., Ledoux P., Evangelista C., Kim I.F., Tomashevsky M., Marshall K.A., Phillippy K.H., Sherman P.M. et al. NCBI GEO: archive for functional genomics data sets–10 years on. Nucleic Acids Res. 2011; 39:D1005–D1010. 21097893 10.1093/nar/gkq1184 PMC3013736

[46] Consortium, E.P. An integrated encyclopedia of DNA elements in the human genome. Nature. 2012; 489:57–74. 22955616 10.1038/nature11247 PMC3439153

[47] Bernstein B.E., Stamatoyannopoulos J.A., Costello J.F., Ren B., Milosavljevic A., Meissner A., Kellis M., Marra M.A., Beaudet A.L., Ecker J.R. et al. The NIH roadmap epigenomics mapping consortium. Nat. Biotechnol. 2010; 28:1045–1048. 20944595 10.1038/nbt1010-1045 PMC3607281

[48] Loven J., Hoke H.A., Lin C.Y., Lau A., Orlando D.A., Vakoc C.R., Bradner J.E., Lee T.I., Young R.A. Selective inhibition of tumor oncogenes by disruption of super-enhancers. Cell. 2013; 153:320–334. 23582323 10.1016/j.cell.2013.03.036 PMC3760967

[49] Langmead B., Trapnell C., Pop M., Salzberg S.L. Ultrafast and memory-efficient alignment of short DNA sequences to the human genome. Genome Biol. 2009; 10:R25. 19261174 10.1186/gb-2009-10-3-r25 PMC2690996

[50] Dibobes G.K., Bol'shakova T.D. [A method of determining vanilmandelic and homovanilic acids by paper chromatography]. Lab. Delo. 1972; 4:221–223. 4119843

[51] Zhang Y., Liu T., Meyer C.A., Eeckhoute J., Johnson D.S., Bernstein B.E., Nusbaum C., Myers R.M., Brown M., Li W. et al. Model-based analysis of ChIP-Seq (MACS). Genome Biol. 2008; 9:R137. 18798982 10.1186/gb-2008-9-9-r137 PMC2592715

[52] Jiang Y., Qian F., Bai X., Liu Y., Wang Q., Ai B., Han X., Shi S., Zhang J., Li X. et al. SEdb: a comprehensive human super-enhancer database. Nucleic Acids Res. 2019; 47:D235–D243. 30371817 10.1093/nar/gky1025 PMC6323980

[53] Qian F.C., Li X.C., Guo J.C., Zhao J.M., Li Y.Y., Tang Z.D., Zhou L.W., Zhang J., Bai X.F., Jiang Y. et al. SEanalysis: a web tool for super-enhancer associated regulatory analysis. Nucleic Acids Res. 2019; 47:W248–W255. 31028388 10.1093/nar/gkz302 PMC6602466

[54] Saint-Andre V., Federation A.J., Lin C.Y., Abraham B.J., Reddy J., Lee T.I., Bradner J.E., Young R.A. Models of human core transcriptional regulatory circuitries. Genome Res. 2016; 26:385–396. 26843070 10.1101/gr.197590.115 PMC4772020

[55] Mei S., Qin Q., Wu Q., Sun H., Zheng R., Zang C., Zhu M., Wu J., Shi X., Taing L. et al. Cistrome Data Browser: a data portal for ChIP-Seq and chromatin accessibility data in human and mouse. Nucleic Acids Res. 2017; 45:D658–D662. 27789702 10.1093/nar/gkw983 PMC5210658

[56] Corces M.R., Granja J.M., Shams S., Louie B.H., Seoane J.A., Zhou W., Silva T.C., Groeneveld C., Wong C.K., Cho S.W. et al. The chromatin accessibility landscape of primary human cancers. Science. 2018; 362:eaav1898. 30361341 10.1126/science.aav1898 PMC6408149

[57] Karolchik D., Barber G.P., Casper J., Clawson H., Cline M.S., Diekhans M., Dreszer T.R., Fujita P.A., Guruvadoo L., Haeussler M. et al. The UCSC Genome Browser database: 2014 update. Nucleic Acids Res. 2014; 42:D764–D770. 24270787 10.1093/nar/gkt1168 PMC3964947

[58] Cheneby J., Menetrier Z., Mestdagh M., Rosnet T., Douida A., Rhalloussi W., Bergon A., Lopez F., Ballester B. ReMap 2020: a database of regulatory regions from an integrative analysis of Human and Arabidopsis DNA-binding sequencing experiments. Nucleic Acids Res. 2020; 48:D180–D188. 31665499 10.1093/nar/gkz945 PMC7145625

[59] Oki S., Ohta T., Shioi G., Hatanaka H., Ogasawara O., Okuda Y., Kawaji H., Nakaki R., Sese J., Meno C. ChIP-Atlas: a data-mining suite powered by full integration of public ChIP-seq data. EMBO Rep. 2018; 19:e46255. 30413482 10.15252/embr.201846255 PMC6280645

[60] Yevshin I., Sharipov R., Valeev T., Kel A., Kolpakov F. GTRD: a database of transcription factor binding sites identified by ChIP-seq experiments. Nucleic Acids Res. 2017; 45:D61–D67. 27924024 10.1093/nar/gkw951 PMC5210645

[61] Quinlan A.R., Hall I.M. BEDTools: a flexible suite of utilities for comparing genomic features. Bioinformatics. 2010; 26:841–842. 20110278 10.1093/bioinformatics/btq033 PMC2832824

[62] Nicolai P., Redaelli de Zinis L.O., Tomenzoli D., Barezzani M.G., Bertoni F., Bignardi M., Antonelli A.R. Prognostic determinants in supraglottic carcinoma: univariate and Cox regression analysis. Head Neck. 1997; 19:323–334. 9213111 10.1002/(sici)1097-0347(199707)19:4<323::aid-hed11>3.0.co;2-a

[63] Wang P., Li X., Gao Y., Guo Q., Wang Y., Fang Y., Ma X., Zhi H., Zhou D., Shen W. et al. LncACTdb 2.0: an updated database of experimentally supported ceRNA interactions curated from low- and high-throughput experiments. Nucleic Acids Res. 2019; 47:D121–D127. 30476305 10.1093/nar/gky1144 PMC6324071

[64] Yu G., Wang L.G., Han Y., He Q.Y. clusterProfiler: an R package for comparing biological themes among gene clusters. Omics. 2012; 16:284–287. 22455463 10.1089/omi.2011.0118 PMC3339379

[65] Altschul S.F., Gish W., Miller W., Myers E.W., Lipman D.J. Basic local alignment search tool. J. Mol. Biol. 1990; 215:403–410. 2231712 10.1016/S0022-2836(05)80360-2

[66] Carithers L.J., Moore H.M. The Genotype-Tissue Expression (GTEx) project. Biopreserv. Biobanking. 2015; 13:307–308. 10.1089/bio.2015.29031.hmm PMC4692118 26484569

[67] Dey-Rao R., Sinha A.A. Genome-wide transcriptional profiling of chronic cutaneous lupus erythematosus (CCLE) peripheral blood identifies systemic alterations relevant to the skin manifestation. Genomics. 2015; 105:90–100. 25451738 10.1016/j.ygeno.2014.11.004

[68] Wu S.M., Liu H., Huang P.J., Chang I.Y., Lee C.C., Yang C.Y., Tsai W.S., Tan B.C. circlncRNAnet: an integrated web-based resource for mapping functional networks of long or circular forms of noncoding RNAs. GigaScience. 2018; 7:gix118. 10.1093/gigascience/gix118 PMC5765557 29194536

[69] Bhan A., Soleimani M., Mandal S.S. Long noncoding RNA and cancer: a new paradigm. Cancer Res. 2017; 77:3965–3981. 28701486 10.1158/0008-5472.CAN-16-2634 PMC8330958

[70] Niknafs Y.S., Han S., Ma T., Speers C., Zhang C., Wilder-Romans K., Iyer M.K., Pitchiaya S., Malik R., Hosono Y. et al. The lncRNA landscape of breast cancer reveals a role for DSCAM-AS1 in breast cancer progression. Nat. Commun. 2016; 7:12791. 27666543 10.1038/ncomms12791 PMC5052669

[71] Lian Y., Xu Y., Xiao C., Xia R., Gong H., Yang P., Chen T., Wu D., Cai Z., Zhang J. et al. The pseudogene derived from long non-coding RNA DUXAP10 promotes colorectal cancer cell growth through epigenetically silencing of p21 and PTEN. Sci. Rep. 2017; 7:7312. 28779166 10.1038/s41598-017-07954-7 PMC5544748

[72] Marzi L., Sun Y., Huang S.N., James A., Difilippantonio S., Pommier Y. The indenoisoquinoline LMP517: a novel antitumor agent targeting both TOP1 and TOP2. Mol. Cancer Ther. 2020; 19:1589–1597. 32430490 10.1158/1535-7163.MCT-19-1064 PMC7415565

[73] Lee Y.J., Ho S.R., Graves J.D., Xiao Y., Huang S., Lin W.C. CGRRF1, a growth suppressor, regulates EGFR ubiquitination in breast cancer. Breast Cancer Res. 2019; 21:134. 31801577 10.1186/s13058-019-1212-2 PMC6894136

[74] Das A., Martinez Santos J.L., Alshareef M., Porto G.B.F., Infinger L.K., Vandergrift W.A. 3rd, Lindhorst S.M., Varma A.K., Patel S.J., Cachia D. In vitro effect of dovitinib (TKI258), a multi-target angiokinase inhibitor on aggressive meningioma cells. Cancer Invest. 2020; 38:349–355. 32441531 10.1080/07357907.2020.1773844

[75] Takaku M., Grimm S.A., De Kumar B., Bennett B.D., Wade P.A. Cancer-specific mutation of GATA3 disrupts the transcriptional regulatory network governed by Estrogen Receptor alpha, FOXA1 and GATA3. Nucleic Acids Res. 2020; 48:4756–4768. 32232341 10.1093/nar/gkaa179 PMC7229857

[76] DennisG.Jr., ShermanB.T., HosackD.A., YangJ., GaoW., LaneH.C., LempickiR.A. DAVID: Database for Annotation, Visualization, and Integrated Discovery. Genome Biol.2003; 4:P3.12734009

[77] Liberzon A., Subramanian A., Pinchback R., Thorvaldsdottir H., Tamayo P., Mesirov J.P. Molecular signatures database (MSigDB) 3.0. Bioinformatics. 2011; 27:1739–1740. 21546393 10.1093/bioinformatics/btr260 PMC3106198

[78] Backes C., Khaleeq Q.T., Meese E., Keller A. miEAA: microRNA enrichment analysis and annotation. Nucleic Acids Res. 2016; 44:W110–W116. 27131362 10.1093/nar/gkw345 PMC4987907

[79] Li J., Han X., Wan Y., Zhang S., Zhao Y., Fan R., Cui Q., Zhou Y. TAM 2.0: tool for MicroRNA set analysis. Nucleic Acids Res. 2018; 46:W180–W185. 29878154 10.1093/nar/gky509 PMC6031048

[80] Han J., Shi X., Zhang Y., Xu Y., Jiang Y., Zhang C., Feng L., Yang H., Shang D., Sun Z. et al. ESEA: discovering the dysregulated pathways based on edge set enrichment analysis. Sci. Rep. 2015; 5:13044. 26267116 10.1038/srep13044 PMC4533315

[81] Han J., Liu S., Sun Z., Zhang Y., Zhang F., Zhang C., Shang D., Yang H., Su F., Xu Y. et al. LncRNAs2Pathways: identifying the pathways influenced by a set of lncRNAs of interest based on a global network propagation method. Sci. Rep. 2017; 7:46566. 28425476 10.1038/srep46566 PMC5397852

[82] Han J., Han X., Kong Q., Cheng L. psSubpathway: a software package for flexible identification of phenotype-specific subpathways in cancer progression. Bioinformatics. 2020; 36:2303–2305. 31821408 10.1093/bioinformatics/btz894

