# Supplementary material for: LncSEA: a platform for long non-coding RNA related sets and enrichment analysis
Source: Nucleic Acids Res. 2020 Oct 12;49(D1):D969–80. doi: 10.1093/nar/gkaa806 (PMC7778898; doi:10.1093/nar/gkaa806)
Supplement: gkaa806_Supplemental_Files [file gkaa806_supplemental_files.zip › Supplementary Figure 1.pdf]

Supplemetary Figure 1

Comparison of enrichment analysis results of four different groups

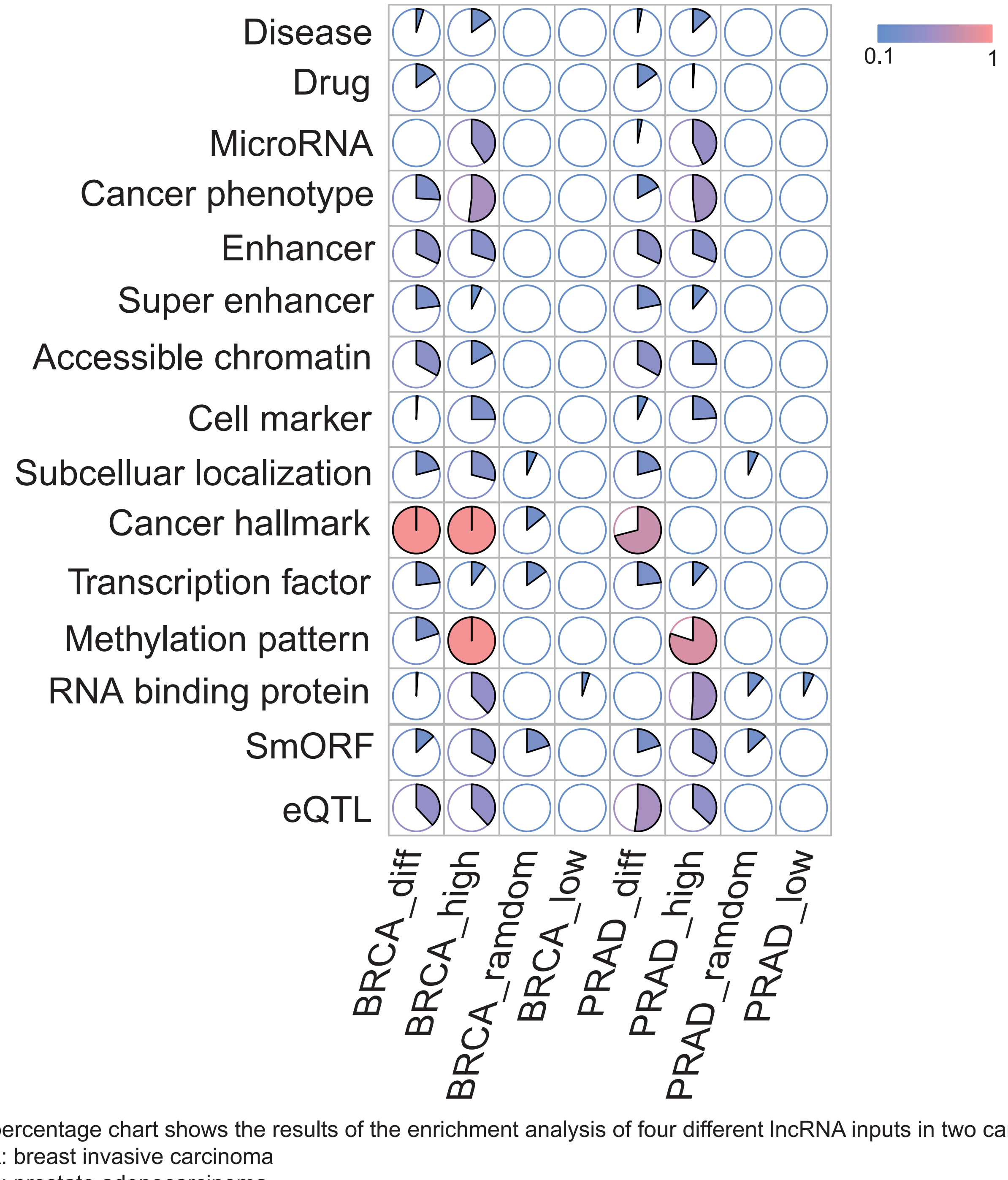

The percentage chart shows the results of the enrichment analysis of four different lncRNA inputs in two cancers.
BRCA: breast invasive carcinoma
PRAD: prostate adenocarcinoma.
'_diff' indicates differentially expressed lncRNAs group .
'_high' indicates high expression lncRNAs group.
'_low' indicates low expression lncRNAs group.
'_random' indicates random sampling of 100 times group.
The number of lncRNAs in each group is the same as the number of differentially expressed lncRNAs group.
Each pie chart represents the number of enriched sets as a percentage of the total number of sets in a category.
The color gradient represents the percentage and the threshold is 0.1-1. The white indicates 0 sets.
