# Supplementary material for: LncSEA: a platform for long non-coding RNA related sets and enrichment analysis
Source: Nucleic Acids Res. 2020 Oct 12;49(D1):D969–80. doi: 10.1093/nar/gkaa806 (PMC7778898; doi:10.1093/nar/gkaa806)
Supplement: gkaa806_Supplemental_Files [file gkaa806_supplemental_files.zip › Supplementary Material 1.docx]

**Supplementary Material 1 Functions comparison LncSEA with others.**

To detail the advantages of LncSEA over other tools, we summarize the differences in six areas:

1. LncSEA contains the largest set of lncRNAs, with more than 40,000 reference sets that are classified into 18 different categories. FARNA only supports disease and a few of functional sets. LnCompare provide more than 100 sets covering six aspects (Supplementary Table 1).
2. Users can browse the details of each reference lncRNA set, including 18 categories, the list of lncRNA names in the set, and the evidence of relationships between the set and each lncRNA in LncSEA. While FARNA provides browse by only three categories. LnCompare is a set analysis web tool that does not support the browsing of set details (Supplementary Table 1).

3) LncSEA supports an interface for conveniently retrieving lncRNA related annotations using multiple names and genomic information. Co-LncRNA and FARNA only support searching using lncRNA names. Lnc-GFP and LnCompare do not provide such functionality (Supplementary Table 1).

4) LncSEA provides upstream and downstream joint enrichment analysis and visualization of results based on more than 40000 reference sets with 18 categories. By contrast, LnCompare provides only 100 sets with 6 aspects for enrichment analysis. Other databases or tools do not support enrichment analysis for lncRNA set (Supplementary Table 1).

5) LncSEA supports the similarity analysis between any two sets of more than 40,000 reference sets to identify relationships between sets in the same category or across 18 different categories. LnCompare also supports similarity analysis (Supplementary Table 1).

6) All reference sets of lncRNAs are arranged and sorted into separate files, which can be downloaded. These sets are organized beforehand into separate files according to the classification, so that users can download them quickly from the website. Others only predict lncRNA functions by online webserver or software and do not support downloading of reference sets.
