# Supplementary material for: LncSEA: a platform for long non-coding RNA related sets and enrichment analysis
Source: Nucleic Acids Res. 2020 Oct 12;49(D1):D969–80. doi: 10.1093/nar/gkaa806 (PMC7778898; doi:10.1093/nar/gkaa806)
Supplement: gkaa806_Supplemental_Files [file gkaa806_supplemental_files.zip › Supplementary Material 2.docx]

**Supplementary Material 2 Materials and Methods**

***C1: Disease*** In recent years, there have been several widely used resources to store the relationship between lncRNAs and diseases. In order to collect sets of diseases, we integrated many experimentally supported associations between lncRNAs and human cancers or diseases from Lnc2Cancer2.0([1](#_ENREF_1)), EVLncRNAs([2](#_ENREF_2)), LncRNADisease([3](#_ENREF_3))2.0 and MNDR2.0 ([4](#_ENREF_4)). We divided the disease category into four sub-categories according to different data sources. Each disease collection consists of a list of lncRNAs associated with the disease. In addition, some supporting evidences like methods and references are also displayed in LncSEA.

***C2: Drug*** We downloaded the relationships between drugs and lncRNAs from LncMap ([5](#_ENREF_5)) database. The relationships were calculated using the Spearman correlations (abs (cor) >0.3, FDR < 0.01) between lncRNA expression levels and IC50 values of 24 drugs across cell lines. The correlation coefficient and P value are displayed in our database. We defined each unique drug related LncRNAs as a predictive set. At the same time, we got a batch of drug-resistant related lncRNAs experimentally supported from Lnc2Cancer2.0 ([1](#_ENREF_1)). In order to distinguish the two sets accurately, we divided the drug set into two subs-categories based on different sources.

***C3: MicroRNA*** A large number of researches showed that lncRNAs performed a variety of regulatory functions for upstream genes such as microRNAs. StarBase2.0 ([6](#_ENREF_6)) and LncBase2.0 ([7](#_ENREF_7)) predicted a large number of miRNA-target interactions using large-scale CLIP-Seq data and algorithms including miRanda and DIANA-microT. Through integrating miRNA-target interactions from StarBase2.0 and LncBase2.0, we defined each unique miRNA related lncRNAs from one source as a set of "MicroRNA" category. These collections will contribute to understand the mechanisms of lncRNAs. For example, lncRNAs often function as competing endogenous RNAs binding miRNA family members in diseases.

***C4:*** ***Cancer Phenotype*** The discovery of cancer subtypes has become one of the research hotspots in oncology. We downloaded phenotype-specific lncRNAs from Cancer RNA-Seq Nexus([8](#_ENREF_8)) that included 40 cancers and 325 phenotypes. We defined 40 cancer sub-categories. Each phenotype (e.g. breast cancer stage II, ER+ breast cancer and Her2+ breast cancer) set contains a group of differential expression of lncRNAs( t-test method, p < 0.05) between two kinds of organization samples. Average expression of samples and significant P value are available in LncSEA. Our lncRNA sets of the different subtypes can provide the basis for accurate and personalized medicine.

***C5&C6: Enhancer and Super Enhancer*** In order to build the "Enhancer" and "Super Enhancer" categories sets, we collected and processed H3K27ac ChIP-seq data from NCBI GEO/SRA([9](#_ENREF_9)), ENCODE([10](#_ENREF_10)), Roadmap([11](#_ENREF_11)) and GGR (Genomics of Gene Regulation Project). To control normalization and consistency across various data sources, we used the streamlined pipeline of Bowtie-MACS-ROSE, which was developed by Loven et al. ([12](#_ENREF_12)). Raw sequencing reads were aligned to hg19 reference genomes with Bowtie([13](#_ENREF_13)) *(Bowtie (v0.12.9) -e 70 -k 2 -n 2 -m 2 -S -q).* Peaks were called using MACS14([14](#_ENREF_14)) (P < 1e-9) *(MACS14(v1.4.2, P<1e-9) -p 1e-9 -w -S --keep-dup=auto --wig --single-profile --space=50)*, and SE regions were annotated using ROSE([12](#_ENREF_12)) software*(ROSE: python ROSE_main.py -g hg19 -i *******.gff –c *******_input.sort.bam -r *******_cas.sort.bam -o ******* -s 12500)*. Finally, we obtained more than 330,000 SE regions involving 542 cells/tissues that was developed by our group. Based on these enhancers and super-enhancers, we identified the lncRNAs regulated by cell-type-specific enhancers and super enhancers using ROSE software GeneMapper.py program ([12](#_ENREF_12)). The enhancer-associated lncRNA will be classified into "Overlap" when the enhancer region overlaps at least one base with the corresponding lncRNA. It will be classified into "proximal" sub-category when the distance between the enhancer and lncRNA is within the range of 50kb. It will be classified into "closest" sub category when the lncRNA is the closest gene and their distance is within the range of 1000kb. Three different positional relationships including "overlap" , "proximal" and "closest" between enhancers and lncRNAs were supported. In addition, we have constructed multiple sets of closest active lncRNAs with super enhancer identified by CRC Mapper program (26843070) in specific cell type.

***C7: Accessible Chromatin*** The chromatin accessibility data, including DNase-seq and ATAC-seq, are available for hundreds of cell types. For DNase-seq, we collected genomic region data of 292 sample types from ENCODE ([10](#_ENREF_10)), Roadmap ([11](#_ENREF_11)) and Cistrome ([15](#_ENREF_15)). For ATAC-seq, we collected genomic region data of 105 sample types from Cistrome, NCBI and 386 samples in 23 kinds of cancer types from TCGA ([16](#_ENREF_16)) (https://tcga-data.nci.nih.gov/tcga). Such databases calculated a large number of peaks by popular processing pipeline. For example, they first obtained FASTA sequence files and aligned all raw sequencing reads using Bowtie and called peaks using MACS. We used the liftOver tool of UCSC ([17](#_ENREF_17)) to convert genomic locations of these genomic region datasets into hg19 version. The ROSE software GeneMapper program ([12](#_ENREF_12)) was also used to predict chromatin-accessibility-region associated lncRNAs with proximity rules including closest, overlapping and proximal.

***C8: Cell Marker*** One of the most fundamental questions in biology is which types of cells form different tissues and organs in a functionally coordinated fashion([9](#_ENREF_9),[18](#_ENREF_18)). Larger-scale single cell sequencing and biological experimental studies are now rapidly opening up new ways to track this question by revealing substantial cell markers for distinguishing different cell types in tissues. Some lncRNAs are also regarded as cell markers to identify cells. We downloaded lncRNAs that are potential to be cell markers from CellMarker ([18](#_ENREF_18)) database for various cell types in tissues of human. Because of the small number of lncRNAs, we did not divide this category into other smaller sub-categories.

***C9: Subcellular Localization*** There are two main mechanisms of LncRNA: transcriptional and post-transcriptional level regulation. The former plays a regulatory role in the nucleus through the role of nuclear factors, while the latter plays a regulatory role in the cytoplasm through the ceRNA mechanism. We collected lncRNAs with different positions in the cells from RNALocate([19](#_ENREF_19)) which provided a lot of lncRNA location information experimentally confirmed and iLoc-lncRNA([20](#_ENREF_20)) that supported information predicted by binomial distribution approach. We divided subcellular localization collection into two categories by data source and data accuracy.

***C10: Cancer Hallmark*** With the development of high-throughput sequencing technology, a large amount of multi-component molecular data have been generated, which brings opportunities to the research of cancer mechanism and cancer treatment. Analysis of the hallmarks of tumours is beneficial to our research and unknown exploration of tumours. We collected lncRNAs as tumour hallmarkers including apoptosis, invasion, metastasis, migration, prognosis and proliferation from CRlncRNA([21](#_ENREF_21)) database. Some additional proof and information such as cancer type, lncRNA expression level and PMID are also supported in LncSEA.

***C11: Transcription Factor*** We collected transcription factor ChIP-seq data of 467 sample types from ENCODE ([10](#_ENREF_10)), Remap ([22](#_ENREF_22)), Cistrome ([15](#_ENREF_15)), ChIP-Atlas ([23](#_ENREF_23)) (http://chip-atlas.org) and GTRD ([24](#_ENREF_24)). Such databases calculated a large number of peaks by popular processing pipeline. For example, Remap aligned all raw sequencing reads using Bowtie2 (with options–end-to-end –sensitive) and all peaks were called using MACS2 (default thresholds, P-value < 1e-5). We used liftOver tool of UCSC ([17](#_ENREF_17)) to convert ChIP-seq peak data into hg19 version. We further identified the peaks overlapping with transcriptional regulatory regions including super enhancers, promoters and Chromatin accessibility regions of lncRNAs using BEDTools (default parameter at least one base overlapping)([25](#_ENREF_25)). Then, the relationships between transcription factors and lncRNAs were built via many kinds of lncRNA-related regulatory regions, such as promoter and enhancer regions bound by transcription factors. Finally, for each transcription factor, we established lncRNA sets with cells/tissues specific regulatory information.

***C12: Methylation Pattern*** LncRNAs play an important role in some essential epigenetic regulation processes such as DNA methylation. DNA methylation is a fundamental feature of epigenomes that can affect the expression of protein-coding or non-coding transcripts. We got manually curated collection and annotation of experimentally supported lncRNA-DNA methylation associations from Lnc2Meth([26](#_ENREF_26)). They were divided into five patterns of DNA methylation, such as "methylation", "demethylation", "hyper methylation", "hypor methylation" and "differential methylation ".

***C13: RNA binding Protein*** RNA binding proteins (RBPs) mediate RNA maturation, transport, localization, and translation. One RBP may have multiple targets and its expression defects can cause multiple diseases. A number of high-throughput techniques and bioinformatics prediction algorithms have been used in recent years to RNA the protein binding relationship. By integrating relationships between lncRNA and binding proteins that rooted in large-scale CLIP-Seq data from StarBase([6](#_ENREF_6)), RNAInter([27](#_ENREF_27)) and EuRBPDB ([28](#_ENREF_28)), we defined each unique protein related LncRNAs as a set. Our lncRNA sets will contribute to investigate the regulatory landscape of cellular lncRNAs.

***C14:*** ***Survival*** Some survival interacted lncRNAs were predicted by downloading and analyzing lncRNA expression data and clinical data. Univariate Cox regression analysis (p < 0.05) ([29](#_ENREF_29))was used to screen out LncRNA related to prognosis. We defined each cancer survival related LncRNAs as a set in TCGA project. Cox regression coefficients, P values and log rank test P values are displayed on set detail pages of our database for user screening and reference. Our survival sets will inform and guide the study of prognosis and lncRNA expression in cancer patients.

***C15: SmORF*** Recent studies found that there are many short or small open ORF (sORFs or smORFs in the body that can encode small peptide. In addition, it was also found that non-coding genes or non-coding regions such as UTR also contained smORFs encoded functional peptides involved in muscle function regulation and regulation of cell metabolism. First,we obtainted encoding small peptides, which were collected from literature, mass spectrometry (MS) and ribosome profiling data by sorf.org ([30](#_ENREF_30))and SmProt([31](#_ENREF_31)). And we got lncRNA annotation information from GENCODE database. By calculating the intersection regions of small peptides and lncRNAs, we obtain some lncRNAs with the function of encoding small peptides and their specific genomic locations. Finally, we classify these sets into several subsets according to the type of lncRNA.

***C16: eQTL*** Numerous studies indicate that lncRNAs have critical functions across biological processes, and single nucleotide polymorphisms (SNPs) could contribute to diseases or traits through influencing lncRNA expression. We aim to build some sets of lncRNAs that have specific mutations in in different cancer types. We collected eQTL−lncRNA pairs caiculated by MatrixEQTL at FDR < 0.05 in 33 cancer types from ncRNA-eQTL([32](#_ENREF_32)) database across 33 cancer types. Both cis- and trans- eQTL studies were included. We also linked ncRNA-eQTLs to genome-wide association study (GWAS) data. Furthermore, we obtained lncRNA sets with four different types including "Common cis" (SNPs affect local ncRNA gene expression) and "Common trans" (SNPs affect distant ncRNA gene expression), "GWAS cis" (eQTLs that overlap with GWAS linkage disequilibrium regions) and "GWAS trans". Further details about SNP are also displayed on the set detail pages of the LncSEA.

***C17:*** ***Exosome*** Exosome is a kind of vesicle secreted by cells. It belongs to an extra cellular vesicle and contains different kinds of RNA to regulate the behavior of receptor cells. It can also be used as a circulating biomarker of disease. We obtained lncRNAs in human blood exosomes with experimental validations in literature from exoRBase ([33](#_ENREF_33)) database to construct an exosome associated lncRNA set.

***C18:*** ***Conservation*** Recent advanced studies have revealed many lncRNAs do not show the same pattern of high interspecies conservation as protein-coding genes ([34](#_ENREF_34)). In order to construct lncRNA sets associated conservation, we obtained the evolutionary conservation of exons and promoters of lncRNAs from LnCompare ([35](#_ENREF_35)), which were calculated using phastCons elements based on multispecies alignment model phylo-HMM. According to the conservative score of each lncRNA, we sorted and divided all the lncRNAs into three sets conservative high (score > 0.65), middle(0.3 < score < 0.65) and low(0.1 < score < 0.3). A conservation category was classified to three sub categories including ‘100 vertebrates" (conservative level across 100 vertebrates), "20 mammals"(conservative level across 20 mammals) and ‘7 vertebrates"(conservative level across 7 vertebrates). These functional sets of conservation can contribute to the functional interpretation of lncRNA.

**Reference**

1. Gao, Y., Wang, P., Wang, Y., Ma, X., Zhi, H., Zhou, D., Li, X., Fang, Y., Shen, W., Xu, Y. *et al.* (2019) Lnc2Cancer v2.0: updated database of experimentally supported long non-coding RNAs in human cancers. *Nucleic acids research*, **47**, D1028-D1033.

2. Zhou, B., Zhao, H., Yu, J., Guo, C., Dou, X., Song, F., Hu, G., Cao, Z., Qu, Y., Yang, Y. *et al.* (2018) EVLncRNAs: a manually curated database for long non-coding RNAs validated by low-throughput experiments. *Nucleic acids research*, **46**, D100-D105.

3. Bao, Z., Yang, Z., Huang, Z., Zhou, Y., Cui, Q. and Dong, D. (2019) LncRNADisease 2.0: an updated database of long non-coding RNA-associated diseases. *Nucleic acids research*, **47**, D1034-D1037.

4. Cui, T., Zhang, L., Huang, Y., Yi, Y., Tan, P., Zhao, Y., Hu, Y., Xu, L., Li, E. and Wang, D. (2018) MNDR v2.0: an updated resource of ncRNA-disease associations in mammals. *Nucleic acids research*, **46**, D371-D374.

5. Li, Y., Li, L., Wang, Z., Pan, T., Sahni, N., Jin, X., Wang, G., Li, J., Zheng, X., Zhang, Y. *et al.* (2018) LncMAP: Pan-cancer atlas of long noncoding RNA-mediated transcriptional network perturbations. *Nucleic acids research*, **46**, 1113-1123.

6. Li, J.H., Liu, S., Zhou, H., Qu, L.H. and Yang, J.H. (2014) starBase v2.0: decoding miRNA-ceRNA, miRNA-ncRNA and protein-RNA interaction networks from large-scale CLIP-Seq data. *Nucleic acids research*, **42**, D92-97.

7. Paraskevopoulou, M.D., Vlachos, I.S., Karagkouni, D., Georgakilas, G., Kanellos, I., Vergoulis, T., Zagganas, K., Tsanakas, P., Floros, E., Dalamagas, T. *et al.* (2016) DIANA-LncBase v2: indexing microRNA targets on non-coding transcripts. *Nucleic acids research*, **44**, D231-238.

8. Li, J.R., Sun, C.H., Li, W., Chao, R.F., Huang, C.C., Zhou, X.J. and Liu, C.C. (2016) Cancer RNA-Seq Nexus: a database of phenotype-specific transcriptome profiling in cancer cells. *Nucleic acids research*, **44**, D944-951.

9. Barrett, T., Troup, D.B., Wilhite, S.E., Ledoux, P., Evangelista, C., Kim, I.F., Tomashevsky, M., Marshall, K.A., Phillippy, K.H., Sherman, P.M. *et al.* (2011) NCBI GEO: archive for functional genomics data sets--10 years on. *Nucleic acids research*, **39**, D1005-1010.

10. Consortium, E.P. (2012) An integrated encyclopedia of DNA elements in the human genome. *Nature*, **489**, 57-74.

11. Bernstein, B.E., Stamatoyannopoulos, J.A., Costello, J.F., Ren, B., Milosavljevic, A., Meissner, A., Kellis, M., Marra, M.A., Beaudet, A.L., Ecker, J.R. *et al.* (2010) The NIH Roadmap Epigenomics Mapping Consortium. *Nature biotechnology*, **28**, 1045-1048.

12. Loven, J., Hoke, H.A., Lin, C.Y., Lau, A., Orlando, D.A., Vakoc, C.R., Bradner, J.E., Lee, T.I. and Young, R.A. (2013) Selective inhibition of tumor oncogenes by disruption of super-enhancers. *Cell*, **153**, 320-334.

13. Langmead, B., Trapnell, C., Pop, M. and Salzberg, S.L. (2009) Ultrafast and memory-efficient alignment of short DNA sequences to the human genome. *Genome biology*, **10**, R25.

14. Zhang, Y., Liu, T., Meyer, C.A., Eeckhoute, J., Johnson, D.S., Bernstein, B.E., Nusbaum, C., Myers, R.M., Brown, M., Li, W. *et al.* (2008) Model-based analysis of ChIP-Seq (MACS). *Genome biology*, **9**, R137.

15. Zheng, R., Wan, C., Mei, S., Qin, Q., Wu, Q., Sun, H., Chen, C.H., Brown, M., Zhang, X., Meyer, C.A. *et al.* (2019) Cistrome Data Browser: expanded datasets and new tools for gene regulatory analysis. *Nucleic acids research*, **47**, D729-D735.

16. Corces, M.R., Granja, J.M., Shams, S., Louie, B.H., Seoane, J.A., Zhou, W., Silva, T.C., Groeneveld, C., Wong, C.K., Cho, S.W. *et al.* (2018) The chromatin accessibility landscape of primary human cancers. *Science*, **362**.

17. Karolchik, D., Barber, G.P., Casper, J., Clawson, H., Cline, M.S., Diekhans, M., Dreszer, T.R., Fujita, P.A., Guruvadoo, L., Haeussler, M. *et al.* (2014) The UCSC Genome Browser database: 2014 update. *Nucleic acids research*, **42**, D764-770.

18. Zhang, X., Lan, Y., Xu, J., Quan, F., Zhao, E., Deng, C., Luo, T., Xu, L., Liao, G., Yan, M. *et al.* (2019) CellMarker: a manually curated resource of cell markers in human and mouse. *Nucleic acids research*, **47**, D721-D728.

19. Zhang, T., Tan, P., Wang, L., Jin, N., Li, Y., Zhang, L., Yang, H., Hu, Z., Zhang, L., Hu, C. *et al.* (2017) RNALocate: a resource for RNA subcellular localizations. *Nucleic acids research*, **45**, D135-D138.

20. Su, Z.D., Huang, Y., Zhang, Z.Y., Zhao, Y.W., Wang, D., Chen, W., Chou, K.C. and Lin, H. (2018) iLoc-lncRNA: predict the subcellular location of lncRNAs by incorporating octamer composition into general PseKNC. *Bioinformatics*, **34**, 4196-4204.

21. Wang, J., Zhang, X., Chen, W., Li, J. and Liu, C. (2018) CRlncRNA: a manually curated database of cancer-related long non-coding RNAs with experimental proof of functions on clinicopathological and molecular features. *BMC medical genomics*, **11**, 114.

22. Cheneby, J., Menetrier, Z., Mestdagh, M., Rosnet, T., Douida, A., Rhalloussi, W., Bergon, A., Lopez, F. and Ballester, B. (2020) ReMap 2020: a database of regulatory regions from an integrative analysis of Human and Arabidopsis DNA-binding sequencing experiments. *Nucleic acids research*, **48**, D180-D188.

23. Oki, S., Ohta, T., Shioi, G., Hatanaka, H., Ogasawara, O., Okuda, Y., Kawaji, H., Nakaki, R., Sese, J. and Meno, C. (2018) ChIP-Atlas: a data-mining suite powered by full integration of public ChIP-seq data. *EMBO reports*, **19**.

24. Yevshin, I., Sharipov, R., Valeev, T., Kel, A. and Kolpakov, F. (2017) GTRD: a database of transcription factor binding sites identified by ChIP-seq experiments. *Nucleic acids research*, **45**, D61-D67.

25. Quinlan, A.R. and Hall, I.M. (2010) BEDTools: a flexible suite of utilities for comparing genomic features. *Bioinformatics*, **26**, 841-842.

26. Zhi, H., Li, X., Wang, P., Gao, Y., Gao, B., Zhou, D., Zhang, Y., Guo, M., Yue, M., Shen, W. *et al.* (2018) Lnc2Meth: a manually curated database of regulatory relationships between long non-coding RNAs and DNA methylation associated with human disease. *Nucleic acids research*, **46**, D133-D138.

27. Lin, Y., Liu, T., Cui, T., Wang, Z., Zhang, Y., Tan, P., Huang, Y., Yu, J. and Wang, D. (2020) RNAInter in 2020: RNA interactome repository with increased coverage and annotation. *Nucleic acids research*, **48**, D189-D197.

28. Liao, J.Y., Yang, B., Zhang, Y.C., Wang, X.J., Ye, Y., Peng, J.W., Yang, Z.Z., He, J.H., Zhang, Y., Hu, K. *et al.* (2019) EuRBPDB: a comprehensive resource for annotation, functional and oncological investigation of eukaryotic RNA binding proteins (RBPs). *Nucleic acids research*.

29. Nicolai, P., Redaelli de Zinis, L.O., Tomenzoli, D., Barezzani, M.G., Bertoni, F., Bignardi, M. and Antonelli, A.R. (1997) Prognostic determinants in supraglottic carcinoma: univariate and Cox regression analysis. *Head & neck*, **19**, 323-334.

30. Olexiouk, V., Van Criekinge, W. and Menschaert, G. (2018) An update on sORFs.org: a repository of small ORFs identified by ribosome profiling. *Nucleic acids research*, **46**, D497-D502.

31. Hao, Y., Zhang, L., Niu, Y., Cai, T., Luo, J., He, S., Zhang, B., Zhang, D., Qin, Y., Yang, F. *et al.* (2018) SmProt: a database of small proteins encoded by annotated coding and non-coding RNA loci. *Briefings in bioinformatics*, **19**, 636-643.

32. Li, J., Xue, Y., Amin, M.T., Yang, Y., Yang, J., Zhang, W., Yang, W., Niu, X., Zhang, H.Y. and Gong, J. (2019) ncRNA-eQTL: a database to systematically evaluate the effects of SNPs on non-coding RNA expression across cancer types. *Nucleic acids research*.

33. Li, S., Li, Y., Chen, B., Zhao, J., Yu, S., Tang, Y., Zheng, Q., Li, Y., Wang, P., He, X. *et al.* (2018) exoRBase: a database of circRNA, lncRNA and mRNA in human blood exosomes. *Nucleic acids research*, **46**, D106-D112.

34. Johnsson, P., Lipovich, L., Grander, D. and Morris, K.V. (2014) Evolutionary conservation of long non-coding RNAs; sequence, structure, function. *Biochimica et biophysica acta*, **1840**, 1063-1071.

35. Carlevaro-Fita, J., Liu, L., Zhou, Y., Zhang, S., Chouvardas, P., Johnson, R. and Li, J. (2019) LnCompare: gene set feature analysis for human long non-coding RNAs. *Nucleic acids research*, **47**, W523-W529.
