# Supplementary material for: LncSEA: a platform for long non-coding RNA related sets and enrichment analysis
Source: Nucleic Acids Res. 2020 Oct 12;49(D1):D969–80. doi: 10.1093/nar/gkaa806 (PMC7778898; doi:10.1093/nar/gkaa806)
Supplement: gkaa806_Supplemental_Files [file gkaa806_supplemental_files.zip › Supplementary Table 1.docx]

**Supplementary Table1. Comparison of LncSEA with other databases and tools.**

| **Function type** | **Functions** | **LncSEA** | **FARNA** | **Co-LncRNA** | **Lnc-GFP** | **LnCompare** |
| --- | --- | --- | --- | --- | --- | --- |
| **LncRNA sets** | Disease | ✔ | ✔ |  |  |  |
|  | Drug | ✔ |  |  |  |  |
|  | MicroRNA | ✔ |  |  |  |  |
|  | Survival | ✔ |  |  |  |  |
|  | RNA binding protein | ✔ |  |  |  |  |
|  | Transcription factor | ✔ |  |  |  |  |
|  | Transcriptional regulatory elements | ✔ |  |  |  |  |
|  | SmORF | ✔ |  |  |  |  |
|  | Conservation | ✔ |  |  |  | ✔ |
|  | Subcellular Localization | ✔ |  |  |  | ✔ |
|  | Exosome | ✔ |  |  |  |  |
|  | Mutations | ✔ |  |  |  | ✔ |
|  | Cancer Phenotype | ✔ |  |  |  |  |
|  | Methylation Pattern | ✔ |  |  |  |  |
|  | Conservation | ✔ |  |  |  | ✔ |
| **LncRNA search** | Search by multiple names; | ✔ | ✔ | ✔ |  |  |
|  | Search by genomic information; | ✔ |  |  |  |  |
| **LncRNA set browse** | Browse by categories | ✔ | ✔ |  |  |  |
| **LncRNA set analysis** | Enrichment analysis | More than 40000 reference lncRNA sets |  |  |  | More than 100 attributes |
|  | Similar analysis across reference sets | ✔ |  |  |  |  |
| **ID** | ID conversion | ✔ |  |  |  |  |

^a^ LncSEA supports 18 kinds of annotations of lncRNA.

^b^ Users can search lncRNA by 5 kinds of names, genomic region or genomic sequence.

^c^ Users can browse all reference sets by 18 different categories.

^d^ LncSEA provides more than 40000 reference sets for enrichment analysis.

^e^ LncSEA supports similar analysis between any two reference sets.

^f^ LncSEA provides ID conversion function with five different names of lncRNA.

^g^ Transcriptional regulatory elements includes enhancer, super enhancer and accessible chromatin.
