# Supplementary material for: LncSEA: a platform for long non-coding RNA related sets and enrichment analysis
Source: Nucleic Acids Res. 2020 Oct 12;49(D1):D969–80. doi: 10.1093/nar/gkaa806 (PMC7778898; doi:10.1093/nar/gkaa806)
Supplement: gkaa806_Supplemental_Files [file gkaa806_supplemental_files.zip › Supplementary Table 3.pdf]

Supplementary Table 3. Sets collection and data processing

No.	Category	Source	Data type	Method & Software
1	Accessible Chromatin	Cistrome; NCBI; TCGA;	ATAC-seq(BED); DNase-seq(BED);	Bowtie; MACS; Genome version: Hg19; ROSE GeneMapper.py;
2 & 3	Enhancer & Super enhancer	ENCODE; Roadmap; NCBI; GGR;	H3K27ac Chip-seq(FASTA);	Bowtie (v0.12.9) -e 70 -k 2 -n 2 -m 2 -S -q; MACS14(v1.4.2, P<1e-9) -p 1e-9 -w -S --keep-dup=auto --wig --single-profile --space=50; ROSE: python ROSE_main.py -g hg19 -i *******.gff –c *******_input.sort.bam -r *******_cas.sort.bam -o ******* -s 12500;
4	Transcription Factor	ENCODE; Remap; Cistrome; GTRD; ChIP-Atlas;	Transcription Factor Chip-seq(BED);	Bowtie; MACS; BEDTools intersection(default parameter at least one base overlapping); Genome version: Hg19; ROSE GeneMapper.py;
5	Survival	TCGA;	Gene expression; Clinical information;	Univariate Cox regression analysis ($p<0.05$);
6	smORF	sorf.org; SmProt;	Encoding small peptides;	Literature; mass spectrometry (MS); ribosome profiling data; BEDTools intersection; Genome version: Hg19;
7	Cancer Hallmark	CRlncRNA;	lncRNA lists;	Literature;
8	Cancer Phenotype	Cancer RNA-Seq Nexus;	Phenotype information;	t-test between two subsets ($p<0.05$);
9	Cell Marker	CellMarker;	lncRNA lists;	Literature; Single cell RNA-seq sequence;
10	Disease	LncRNADisease2.0; MNDR2.0; Lnc2Cancer2.0; EVLncRNAs;	Relationship of lncRNAs and diseases;	Literature;
11	Drug	Lnc2Cancer2.0; LncMap;	Relationship of lncRNAs and drugs;	Literature; Spearman correlation (abs(cor) >0.3, FDR <0.01);
12	EQTL	ncRNA-eQTL;	Mutations of lncRNA;	IMPUTE2; EIGENSOFT; PEER; MatrixEQTL;
13	Methylation Pattern	Lnc2Meth;	Methylation of lncRNA;	Literature;
14	MicroRNA	StarBase2.0; LncBase2.0	Relationship of lncRNAs and miRNAs;	Literature; CLIP-seq; miRanda; DIANA-microT;
15	RNA Binding Protein	RNAInter; EuRBPDB; StarBase;	Relationship of lncRNAs and Proteins;	High throughput; Literature; Computational prediction;
16	Subcellular Localization	RNALocate; iLoc-lncRNA;	Subcellular localization of lncRNA;	Literature; Binomial distribution approach;
17	Exosome	exoRBase;	LncRNA list;	Literature;
18	Conservation	LnCompare;	Conservation scores of lncRNAs;	phastCons conservation score;
